# Supplementary material for: Glucagon-like peptide-1 receptor activation stimulates PKA-mediated phosphorylation of Raptor and this contributes to the weight loss effect of liraglutide
Source: eLife. 2023 Nov 6;12:e80944. doi: 10.7554/eLife.80944 (PMC10691799; doi:10.7554/eLife.80944)
Supplement: Figure 1—source data 1. [file elife-80944-fig1-data1.zip › Resubmission Rev 2 Figure 1-source data 1/eLife PKA Manuscript Rev 2 Figure 1 Raw Blots.pptx]

## Slide 1
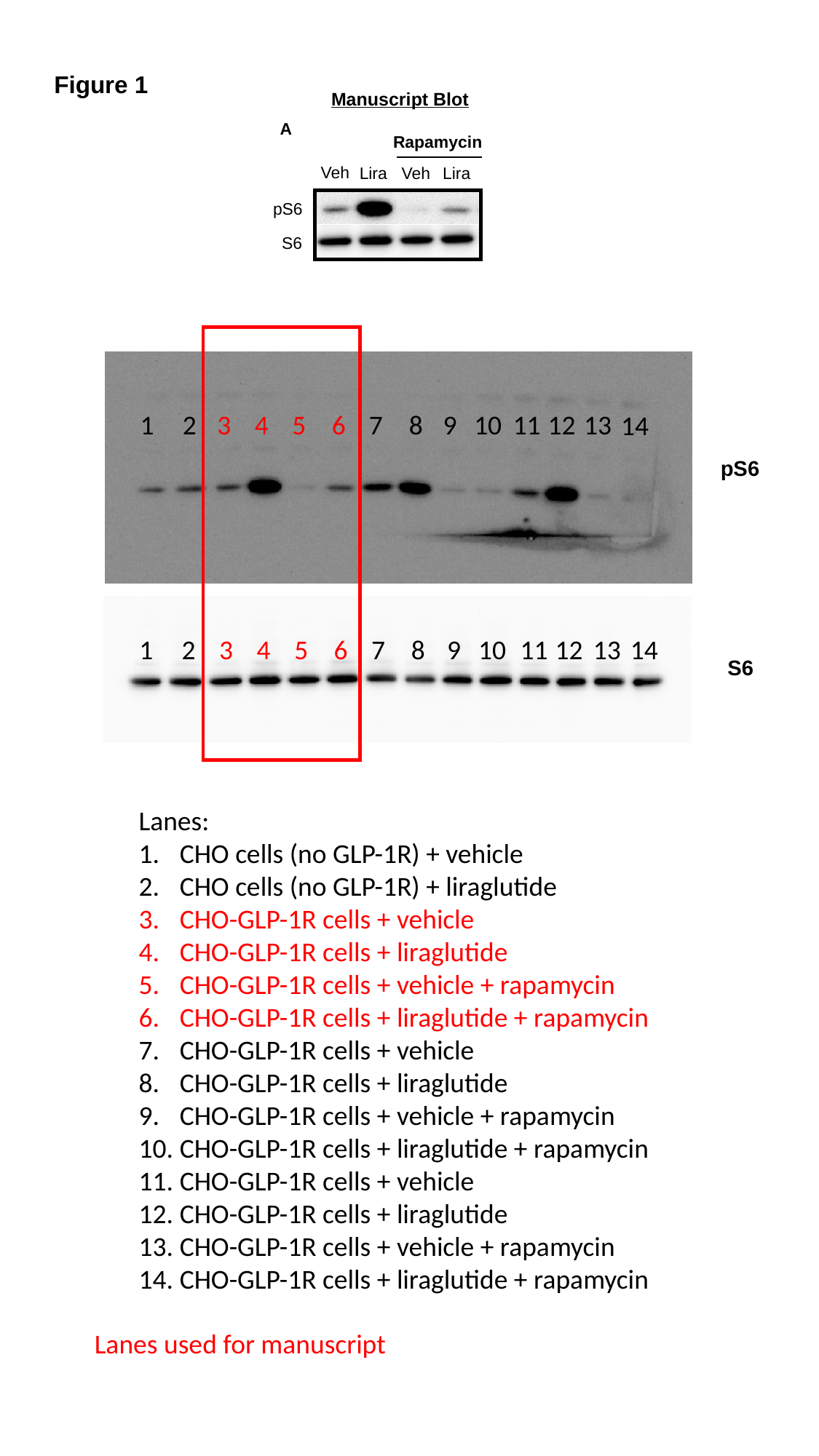

Figure 1
Manuscript Blot
A
Rapamycin
Veh
Veh
Lira
Lira
pS6
S6
1
2
3
4
5
6
7
8
9
10
11
12
13
14
pS6
1
2
3
4
5
6
7
8
9
10
11
12
13
14
S6
Lanes:
CHO cells (no GLP-1R) + vehicle
CHO cells (no GLP-1R) + liraglutide
CHO-GLP-1R cells + vehicle
CHO-GLP-1R cells + liraglutide
CHO-GLP-1R cells + vehicle + rapamycin
CHO-GLP-1R cells + liraglutide + rapamycin
CHO-GLP-1R cells + vehicle
CHO-GLP-1R cells + liraglutide
CHO-GLP-1R cells + vehicle + rapamycin
CHO-GLP-1R cells + liraglutide + rapamycin
CHO-GLP-1R cells + vehicle
CHO-GLP-1R cells + liraglutide
CHO-GLP-1R cells + vehicle + rapamycin
CHO-GLP-1R cells + liraglutide + rapamycin
Lanes used for manuscript

## Slide 2
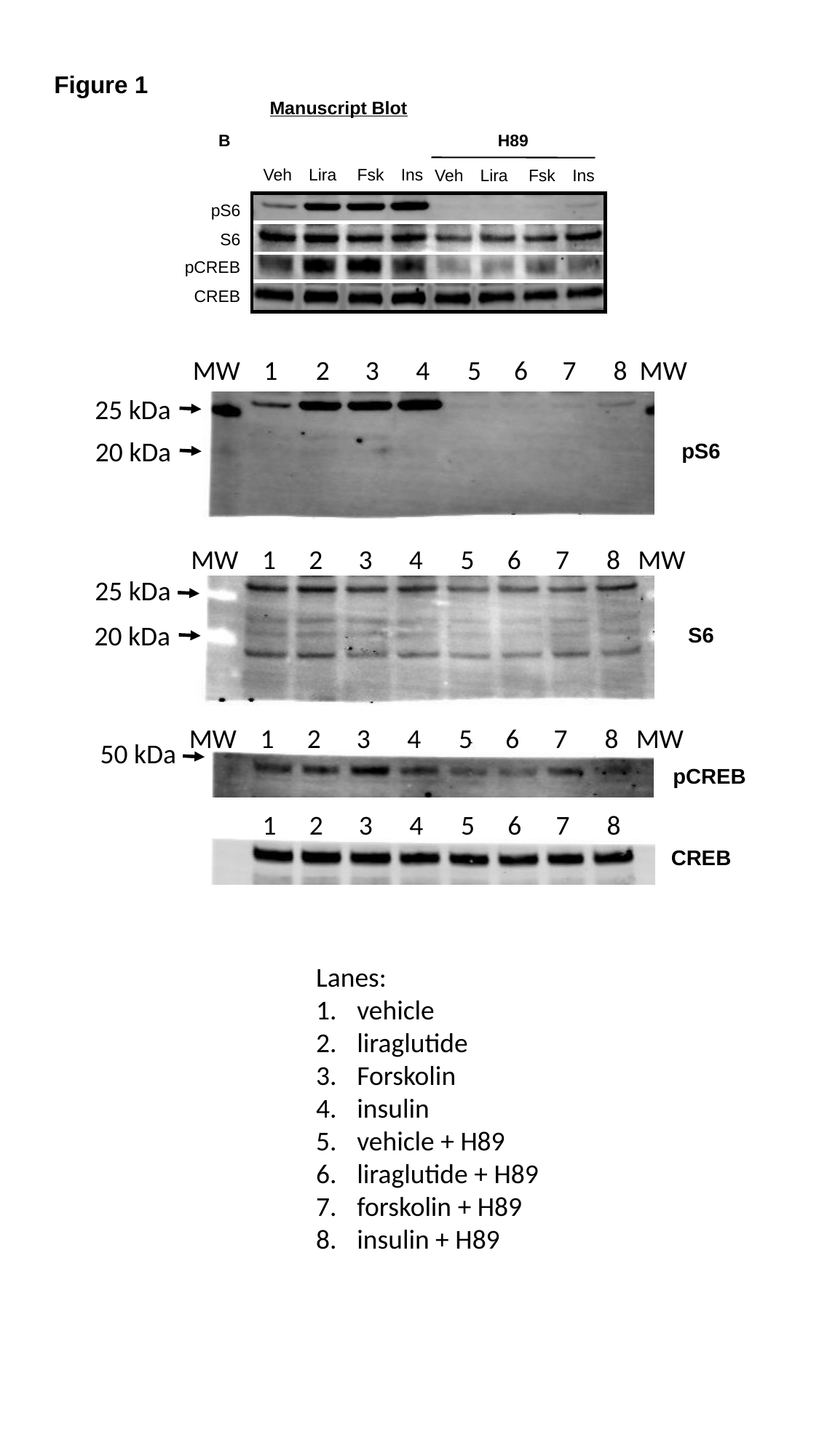

Figure 1
Manuscript Blot
H89
Veh
Lira
Fsk
Ins
Veh
Lira
Fsk
Ins
pS6
S6
pCREB
CREB
B
MW
1
2
3
4
5
6
7
8
MW
25 kDa
20 kDa
pS6
MW
1
2
3
4
5
6
7
8
MW
25 kDa
20 kDa
S6
MW
1
2
3
4
5
6
7
8
MW
50 kDa
pCREB
1
2
3
4
5
6
7
8
CREB
Lanes:
vehicle
liraglutide
Forskolin
insulin
vehicle + H89
liraglutide + H89
forskolin + H89
insulin + H89

## Slide 3
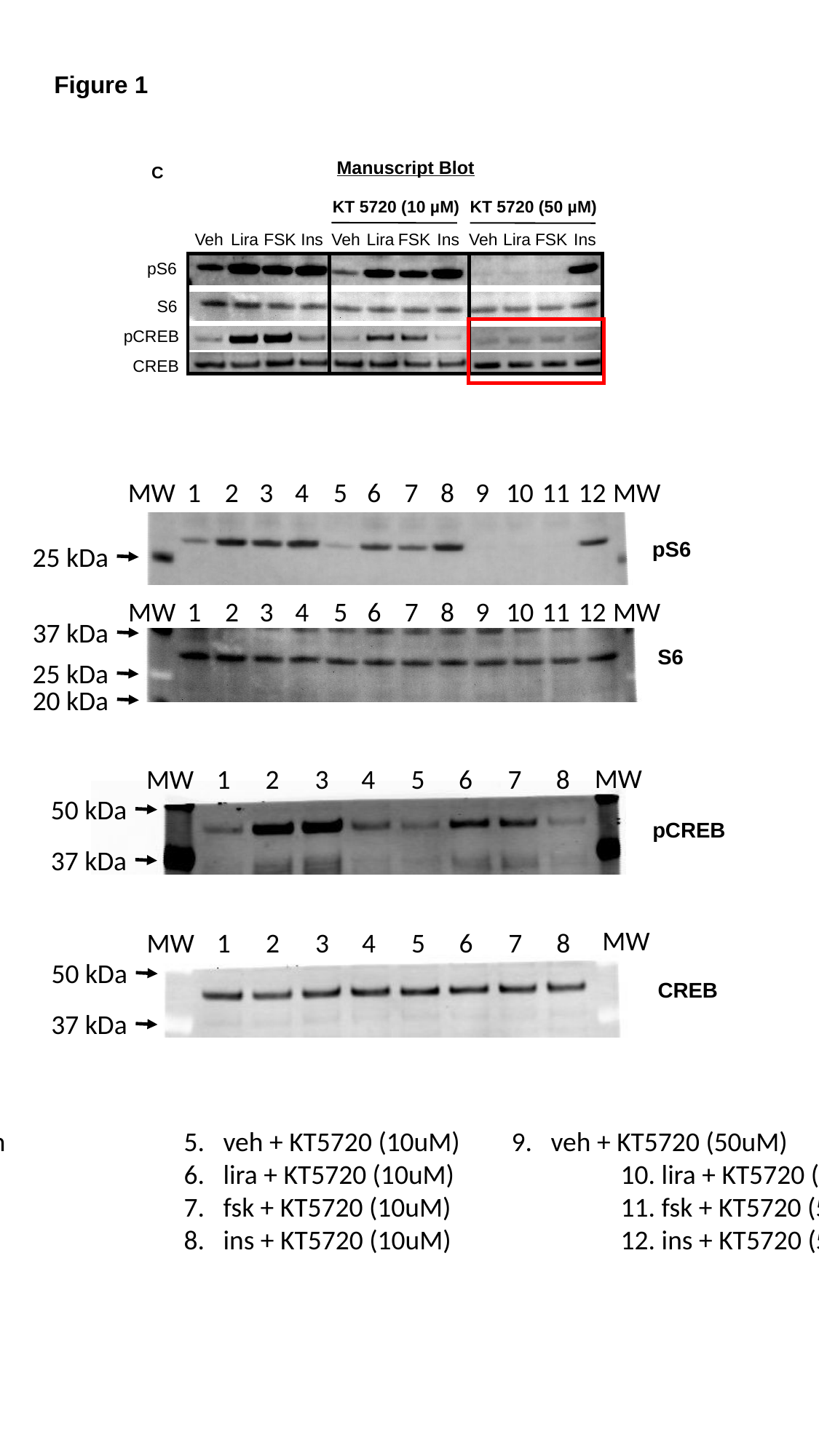

Figure 1
Manuscript Blot
C
KT 5720 (10 µM)
KT 5720 (50 µM)
Veh
Lira
FSK
Ins
Veh
Lira
FSK
Ins
Veh
Lira
FSK
Ins
pS6
S6
pCREB
CREB
MW
1
2
3
4
5
6
7
8
9
10
11
12
MW
pS6
25 kDa
MW
1
2
3
4
5
6
7
8
9
10
11
12
MW
37 kDa
S6
25 kDa
20 kDa
MW
MW
1
2
3
4
5
6
7
8
50 kDa
pCREB
37 kDa
MW
MW
1
2
3
4
5
6
7
8
50 kDa
CREB
37 kDa
Lanes:
veh		5. veh + KT5720 (10uM)	9. veh + KT5720 (50uM)
lira		6. lira + KT5720 (10uM)		10. lira + KT5720 (50uM)
fsk		7. fsk + KT5720 (10uM)		11. fsk + KT5720 (50uM)
ins		8. ins + KT5720 (10uM)		12. ins + KT5720 (50uM)

## Slide 4
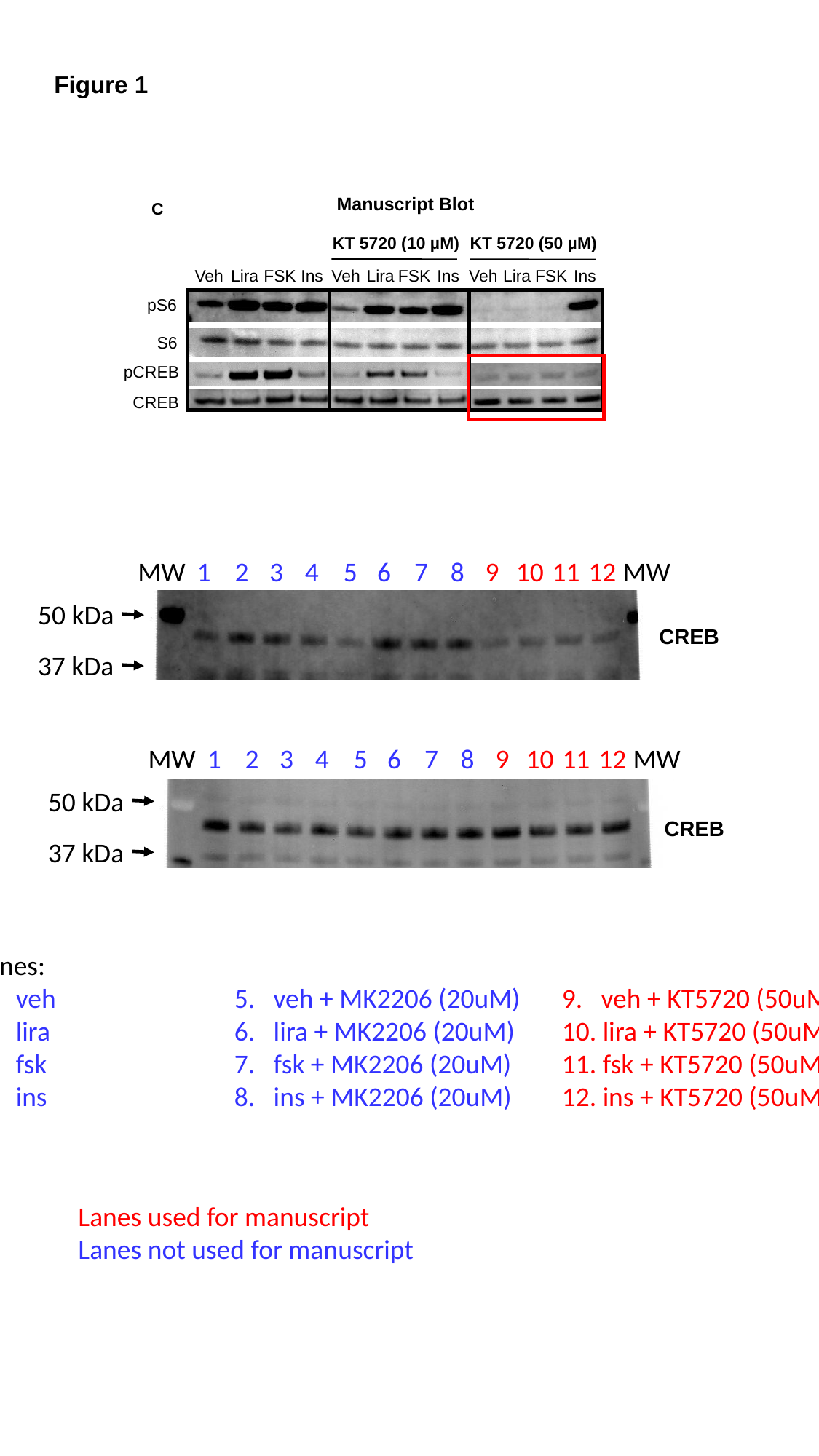

Figure 1
Manuscript Blot
C
KT 5720 (10 µM)
KT 5720 (50 µM)
Veh
Lira
FSK
Ins
Veh
Lira
FSK
Ins
Veh
Lira
FSK
Ins
pS6
S6
pCREB
CREB
MW
1
2
3
4
5
6
7
8
9
10
11
12
MW
50 kDa
CREB
37 kDa
MW
1
2
3
4
5
6
7
8
9
10
11
12
MW
50 kDa
CREB
37 kDa
Lanes:
veh		5. veh + MK2206 (20uM)	9. veh + KT5720 (50uM)
lira		6. lira + MK2206 (20uM)	10. lira + KT5720 (50uM)
fsk		7. fsk + MK2206 (20uM)	11. fsk + KT5720 (50uM)
ins		8. ins + MK2206 (20uM)	12. ins + KT5720 (50uM)
Lanes used for manuscript
Lanes not used for manuscript

## Slide 5
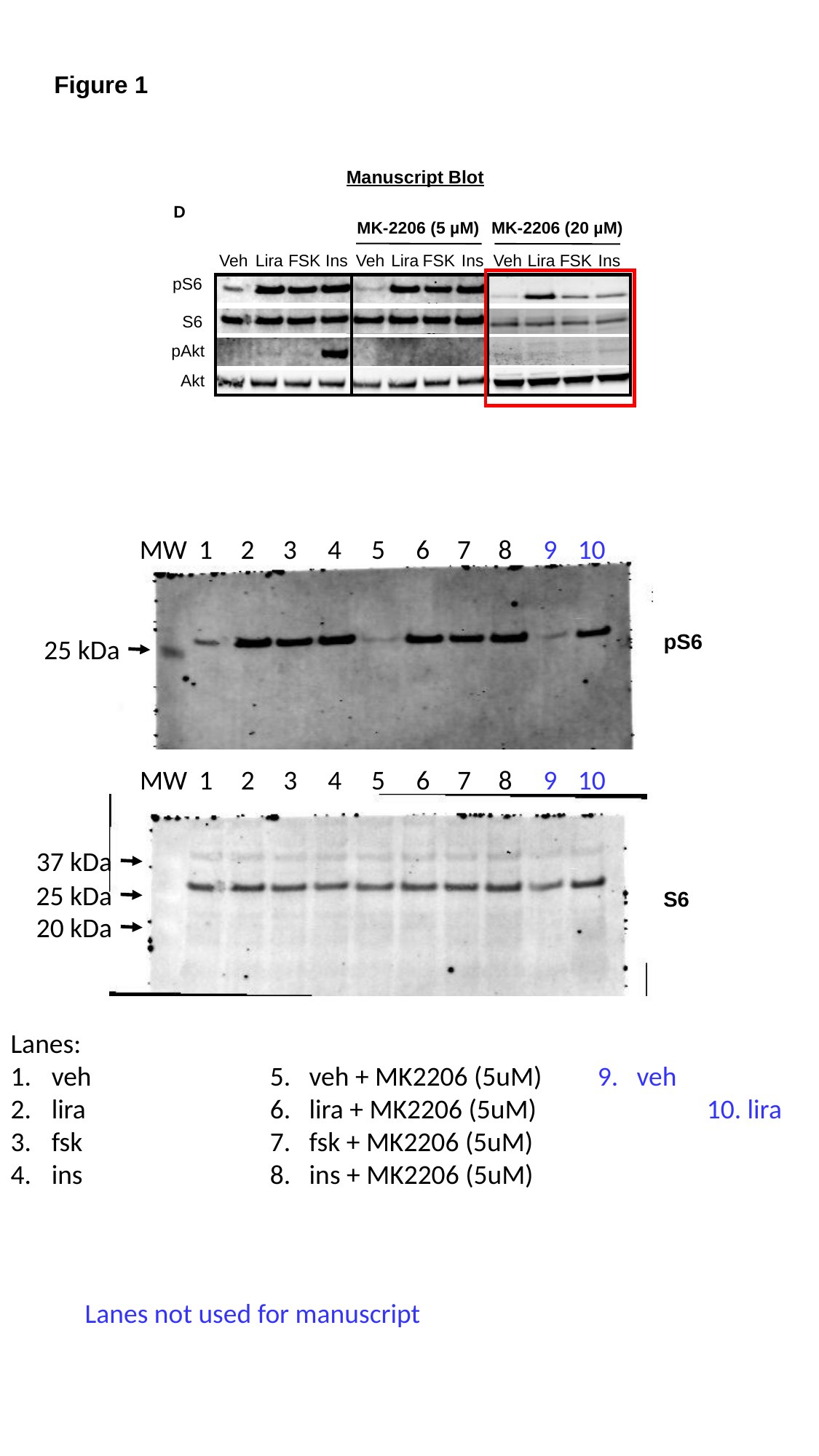

Figure 1
Manuscript Blot
D
MK-2206 (5 µM)
MK-2206 (20 µM)
Veh
Lira
FSK
Ins
Veh
Lira
FSK
Ins
Veh
Lira
FSK
Ins
pS6
S6
pAkt
Akt
MW
1
2
3
4
5
6
7
8
9
10
pS6
25 kDa
MW
1
2
3
4
5
6
7
8
9
10
37 kDa
25 kDa
S6
20 kDa
Lanes:
veh		5. veh + MK2206 (5uM)	9. veh
lira		6. lira + MK2206 (5uM)		10. lira
fsk		7. fsk + MK2206 (5uM)
ins		8. ins + MK2206 (5uM)
Lanes not used for manuscript

## Slide 6
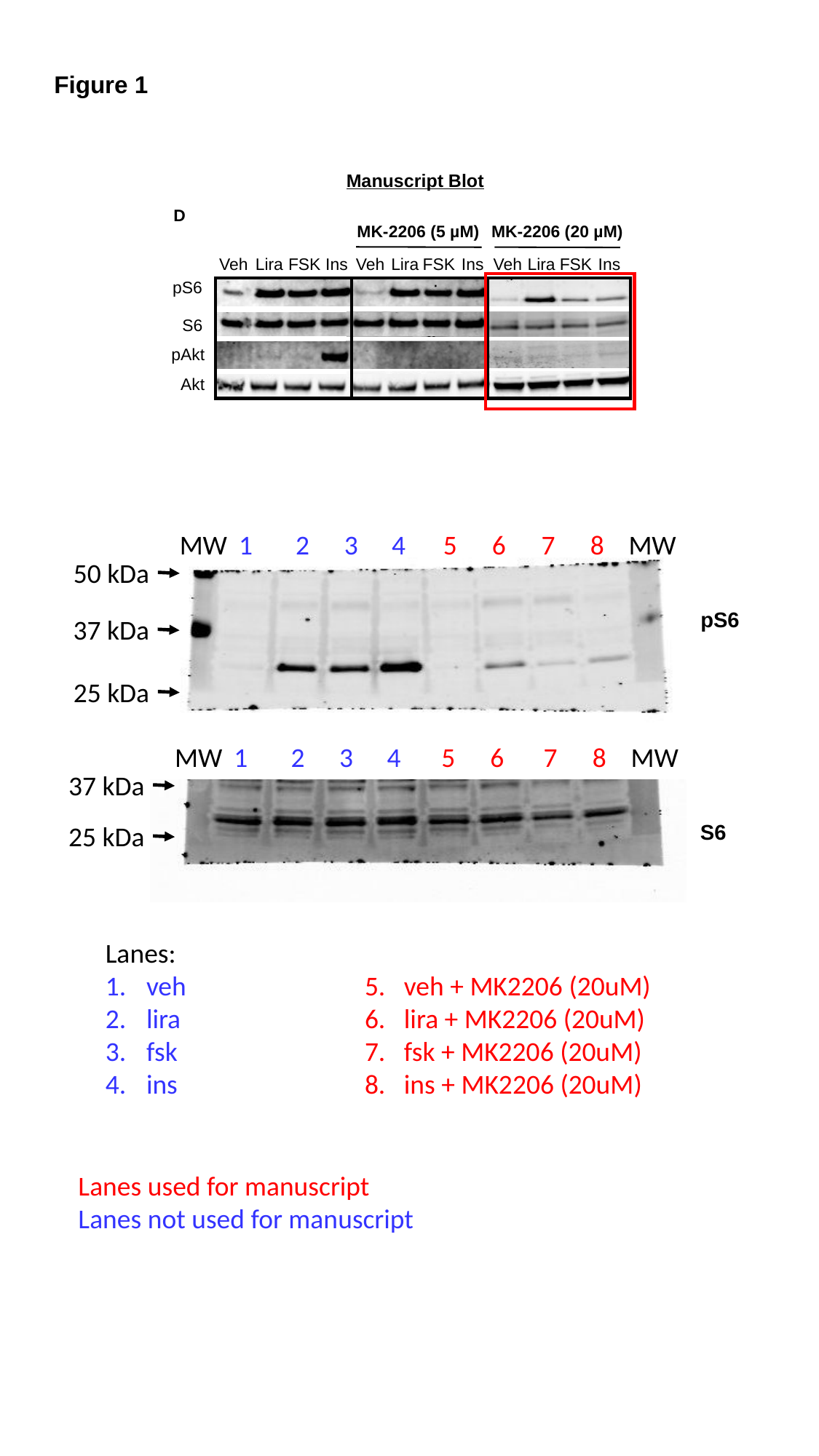

Figure 1
Manuscript Blot
D
MK-2206 (5 µM)
MK-2206 (20 µM)
Veh
Lira
FSK
Ins
Veh
Lira
FSK
Ins
Veh
Lira
FSK
Ins
pS6
S6
pAkt
Akt
MW
1
2
3
4
5
6
7
8
MW
50 kDa
pS6
37 kDa
25 kDa
MW
1
2
3
4
5
6
7
8
MW
37 kDa
S6
25 kDa
Lanes:
veh		5. veh + MK2206 (20uM)
lira		6. lira + MK2206 (20uM)
fsk		7. fsk + MK2206 (20uM)
ins		8. ins + MK2206 (20uM)
Lanes used for manuscript
Lanes not used for manuscript

## Slide 7
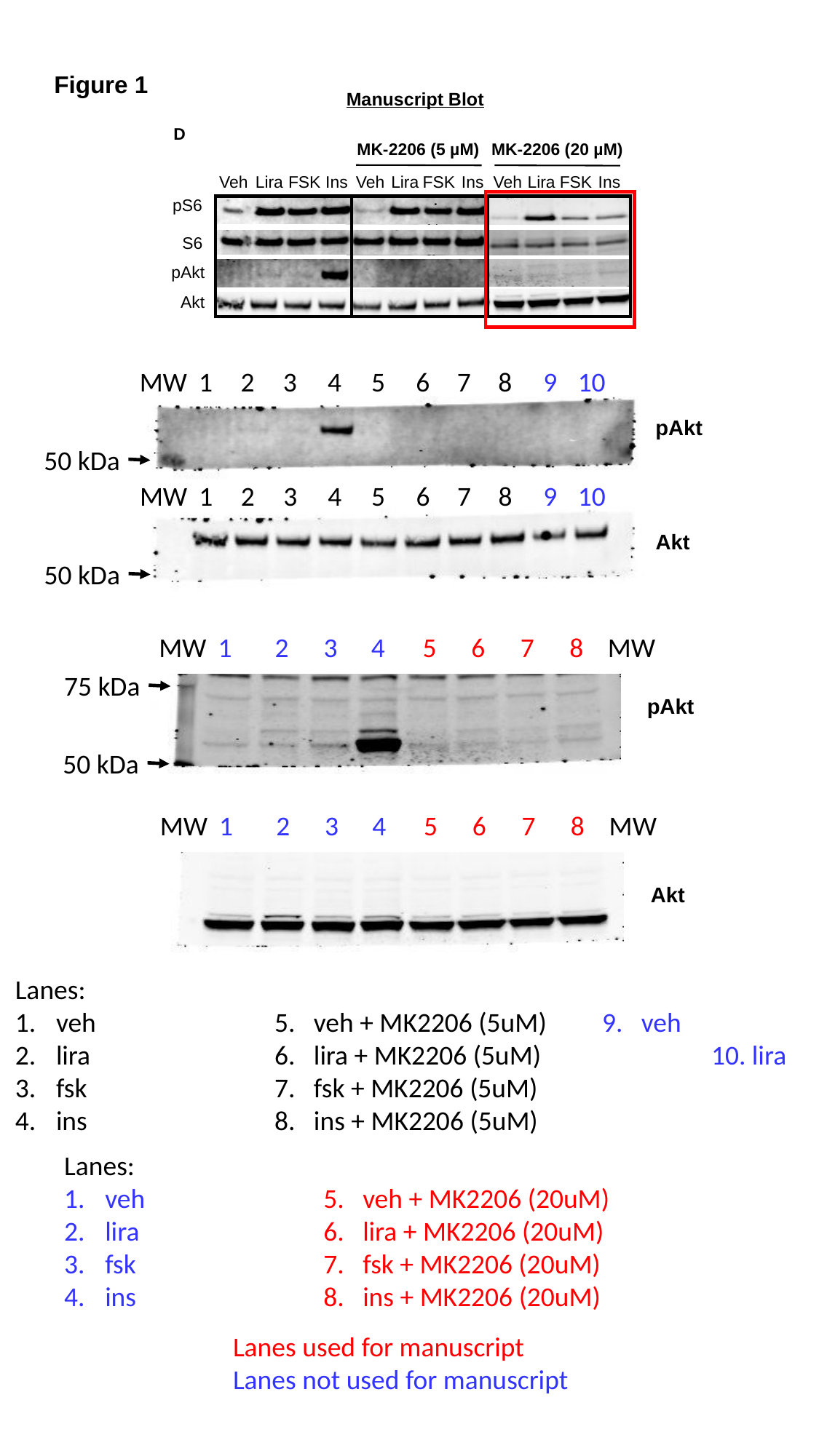

Figure 1
Manuscript Blot
D
MK-2206 (5 µM)
MK-2206 (20 µM)
Veh
Lira
FSK
Ins
Veh
Lira
FSK
Ins
Veh
Lira
FSK
Ins
pS6
S6
pAkt
Akt
MW
1
2
3
4
5
6
7
8
9
10
pAkt
50 kDa
MW
1
2
3
4
5
6
7
8
9
10
Akt
50 kDa
MW
1
2
3
4
5
6
7
8
MW
75 kDa
pAkt
50 kDa
MW
1
2
3
4
5
6
7
8
MW
Akt
Lanes:
veh		5. veh + MK2206 (5uM)	9. veh
lira		6. lira + MK2206 (5uM)		10. lira
fsk		7. fsk + MK2206 (5uM)
ins		8. ins + MK2206 (5uM)
Lanes:
veh		5. veh + MK2206 (20uM)
lira		6. lira + MK2206 (20uM)
fsk		7. fsk + MK2206 (20uM)
ins		8. ins + MK2206 (20uM)
Lanes used for manuscript
Lanes not used for manuscript
