# Supplementary material for: Glucagon-like peptide-1 receptor activation stimulates PKA-mediated phosphorylation of Raptor and this contributes to the weight loss effect of liraglutide
Source: eLife. 2023 Nov 6;12:e80944. doi: 10.7554/eLife.80944 (PMC10691799; doi:10.7554/eLife.80944)
Supplement: Figure 1—source data 1. [file elife-80944-fig1-data1.zip › Resubmission Rev 2 Figure 1-source data 1/eLife PKA Manuscript Rev 2 Figure 1.pptx]

## Slide 1
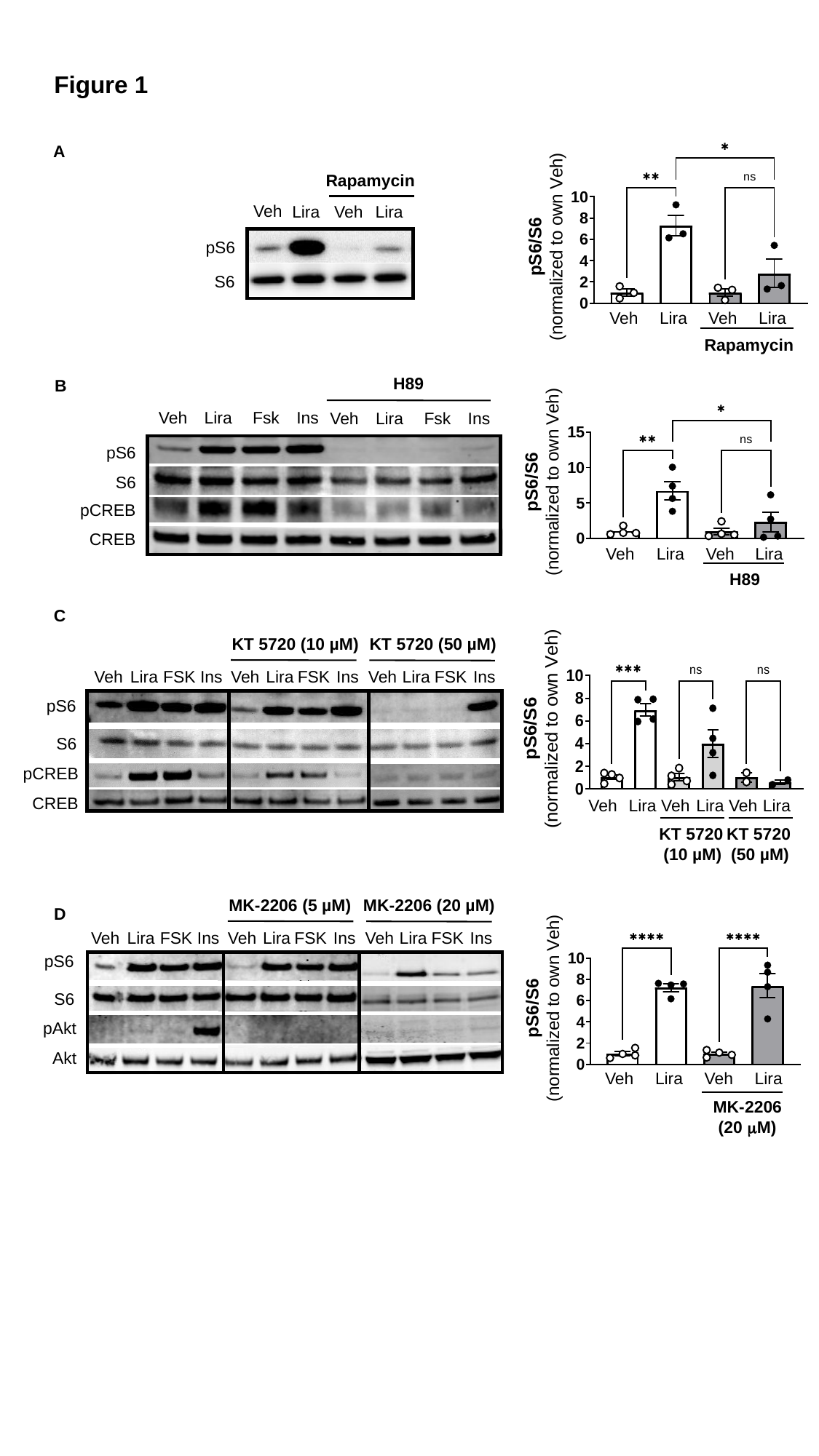

Figure 1
A
Rapamycin
Veh
Veh
Lira
Lira
pS6
S6
Veh
Lira
Veh
Lira
Rapamycin
H89
B
Veh
Lira
Fsk
Ins
Veh
Lira
Fsk
Ins
pS6
S6
pCREB
CREB
Veh
Lira
Veh
Lira
H89
C
KT 5720 (10 µM)
KT 5720 (50 µM)
Veh
Lira
FSK
Ins
Veh
Lira
FSK
Ins
Veh
Lira
FSK
Ins
pS6
S6
pCREB
CREB
Veh
Lira
Veh
Lira
Veh
Lira
KT 5720
(10 µM)
KT 5720
(50 µM)
MK-2206 (5 µM)
MK-2206 (20 µM)
D
Veh
Lira
FSK
Ins
Veh
Lira
FSK
Ins
Veh
Lira
FSK
Ins
pS6
S6
pAkt
Akt
Veh
Lira
Veh
Lira
MK-2206
(20 mM)
