# Supplementary figures and images for: Glucagon-like peptide-1 receptor activation stimulates PKA-mediated phosphorylation of Raptor and this contributes to the weight loss effect of liraglutide
Source: eLife. 2023 Nov 6;12:e80944. doi: 10.7554/eLife.80944 (PMC10691799; doi:10.7554/eLife.80944)

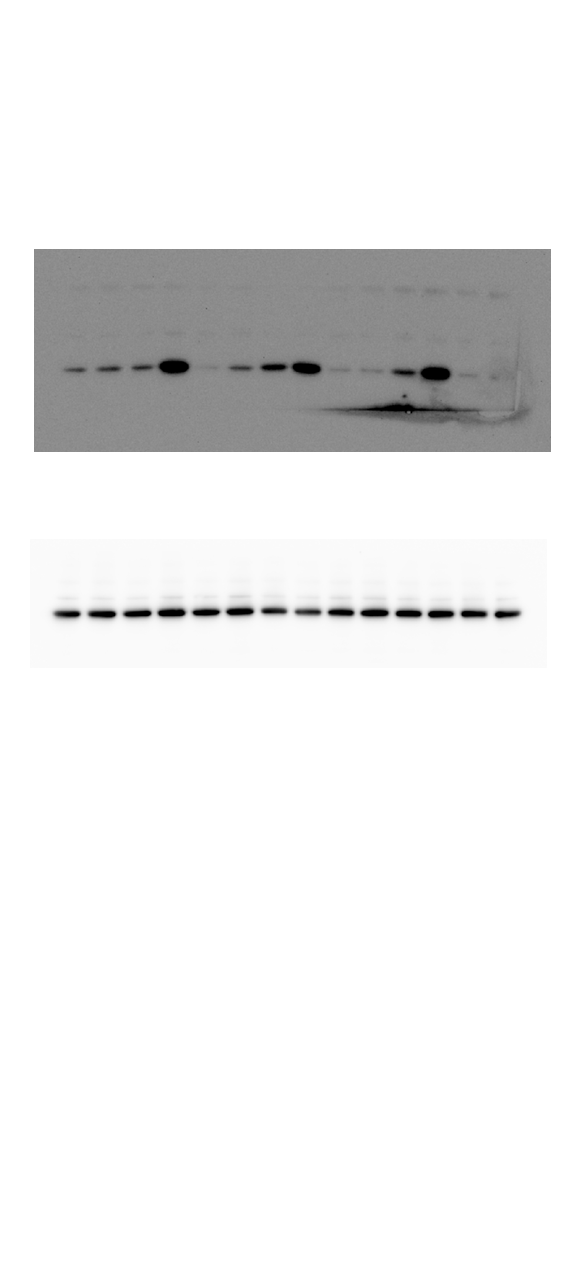

Supplement: Figure 1—source data 1. [file elife-80944-fig1-data1.zip › Resubmission Rev 2 Figure 1-source data 1/Figure 1A Raw Blots.tiff]

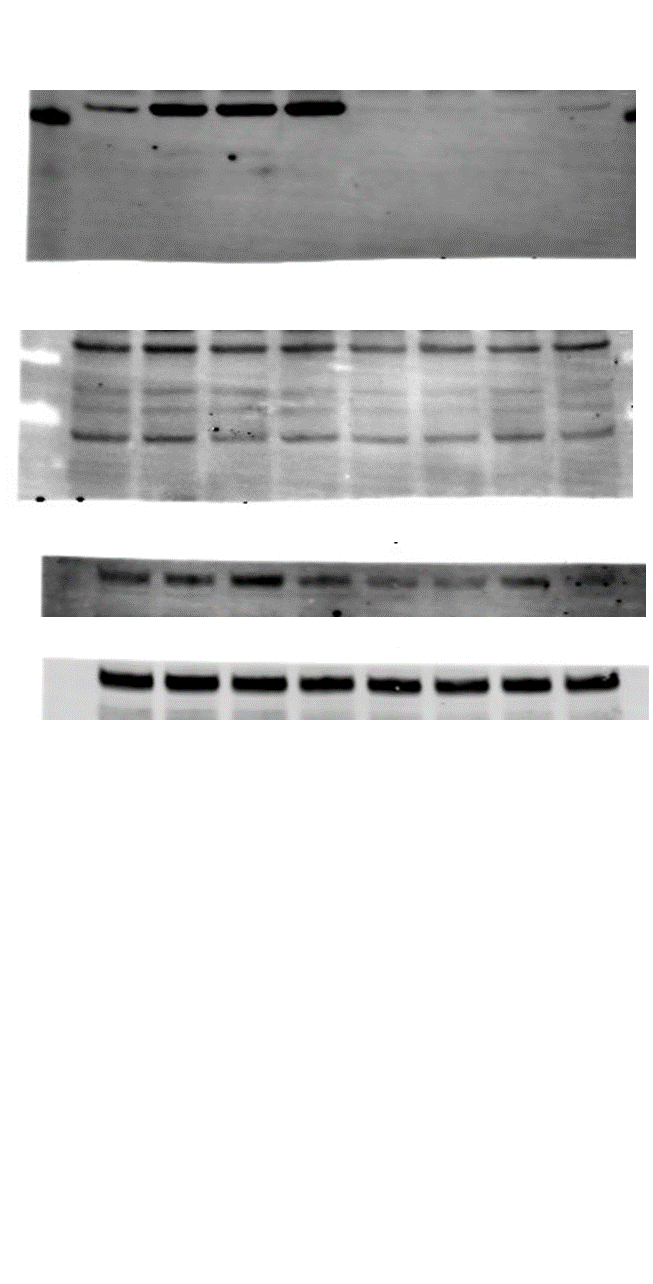

Supplement: Figure 1—source data 1. [file elife-80944-fig1-data1.zip › Resubmission Rev 2 Figure 1-source data 1/Figure 1B Raw Blots.tiff]

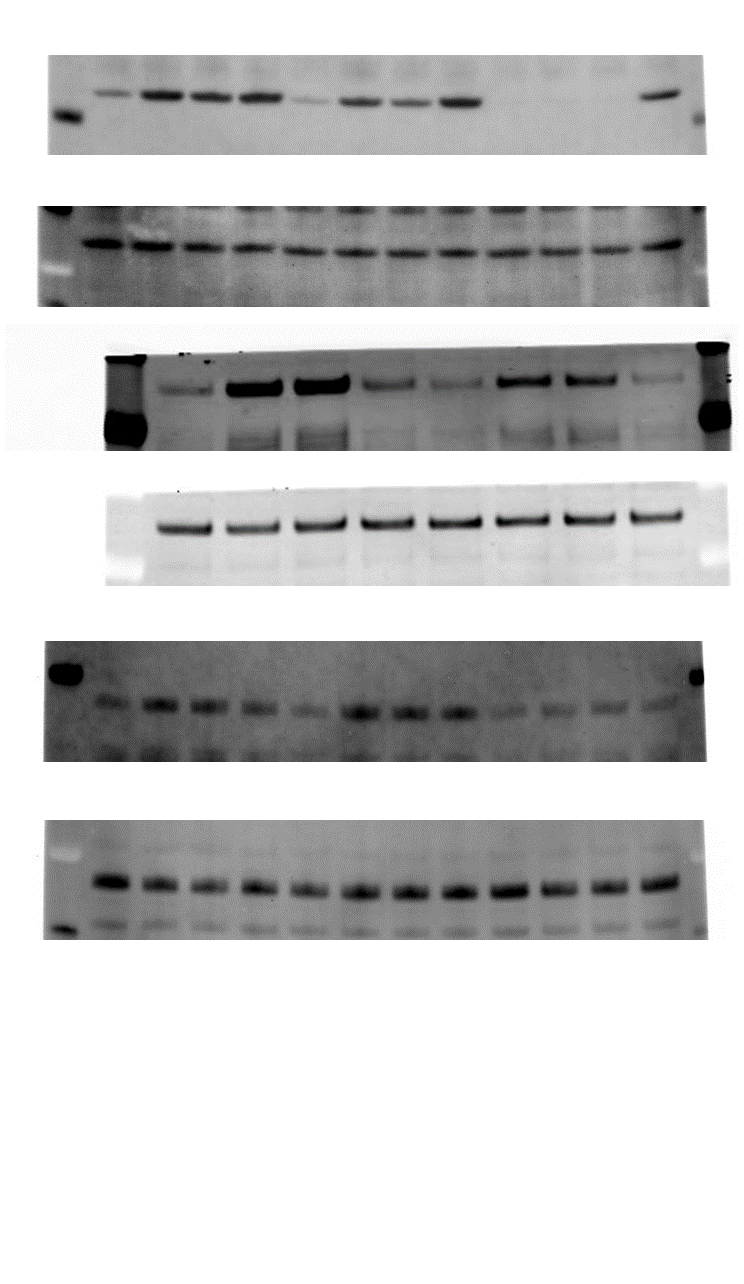

Supplement: Figure 1—source data 1. [file elife-80944-fig1-data1.zip › Resubmission Rev 2 Figure 1-source data 1/Figure 1C Raw Blots.tiff]

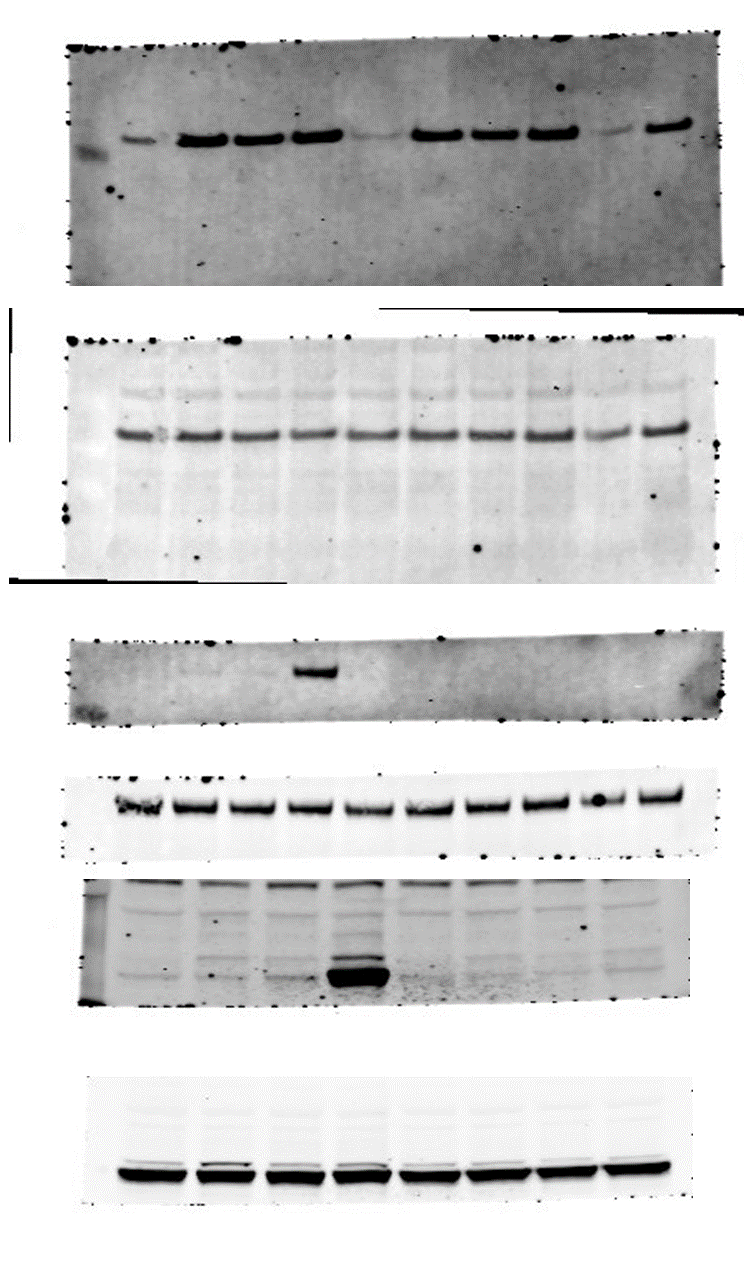

Supplement: Figure 1—source data 1. [file elife-80944-fig1-data1.zip › Resubmission Rev 2 Figure 1-source data 1/Figure 1D Raw Blots.tiff]

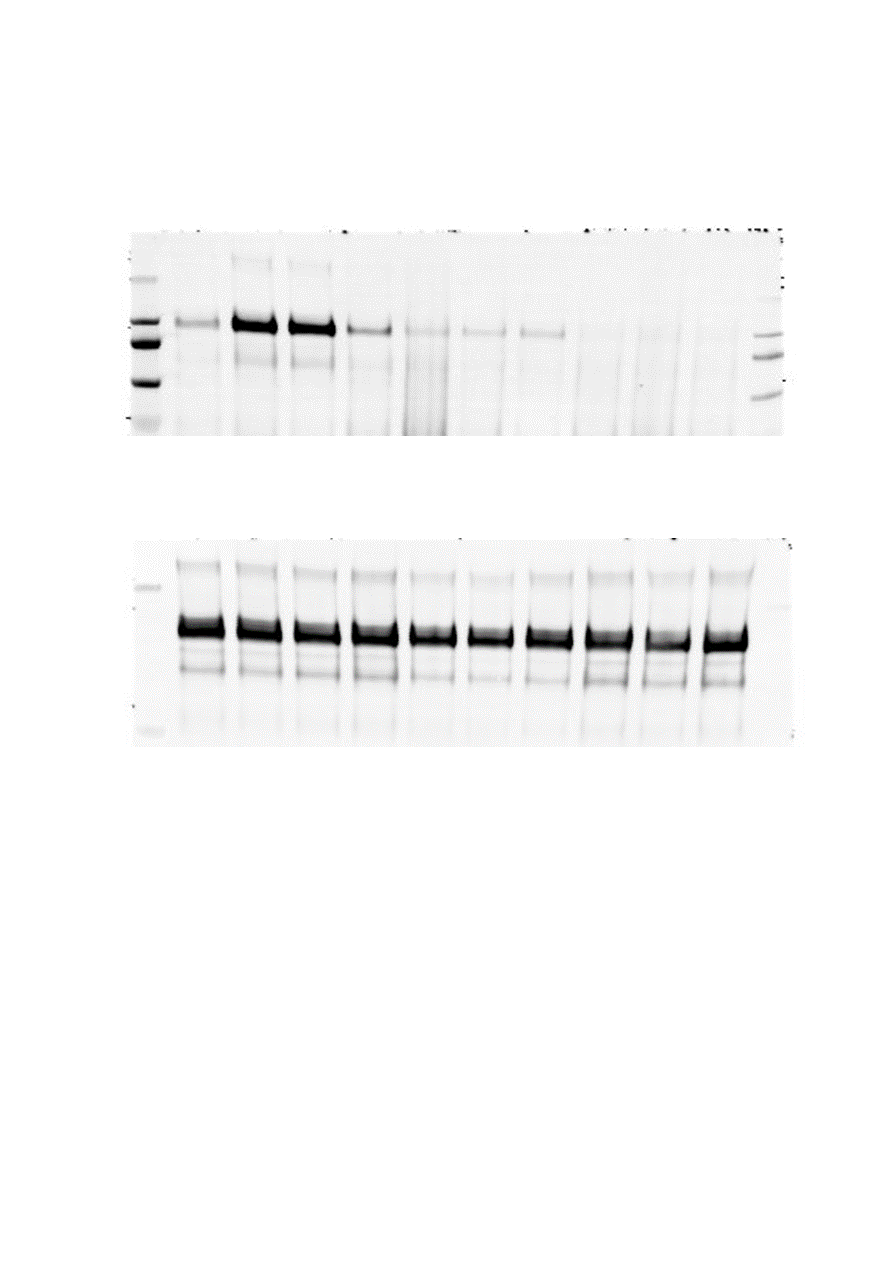

Supplement: Figure 2—source data 1. [file elife-80944-fig2-data1.zip › Resubmission Rev 2 Figure 2source data 1/Figure 2A Raw Blots.tiff]

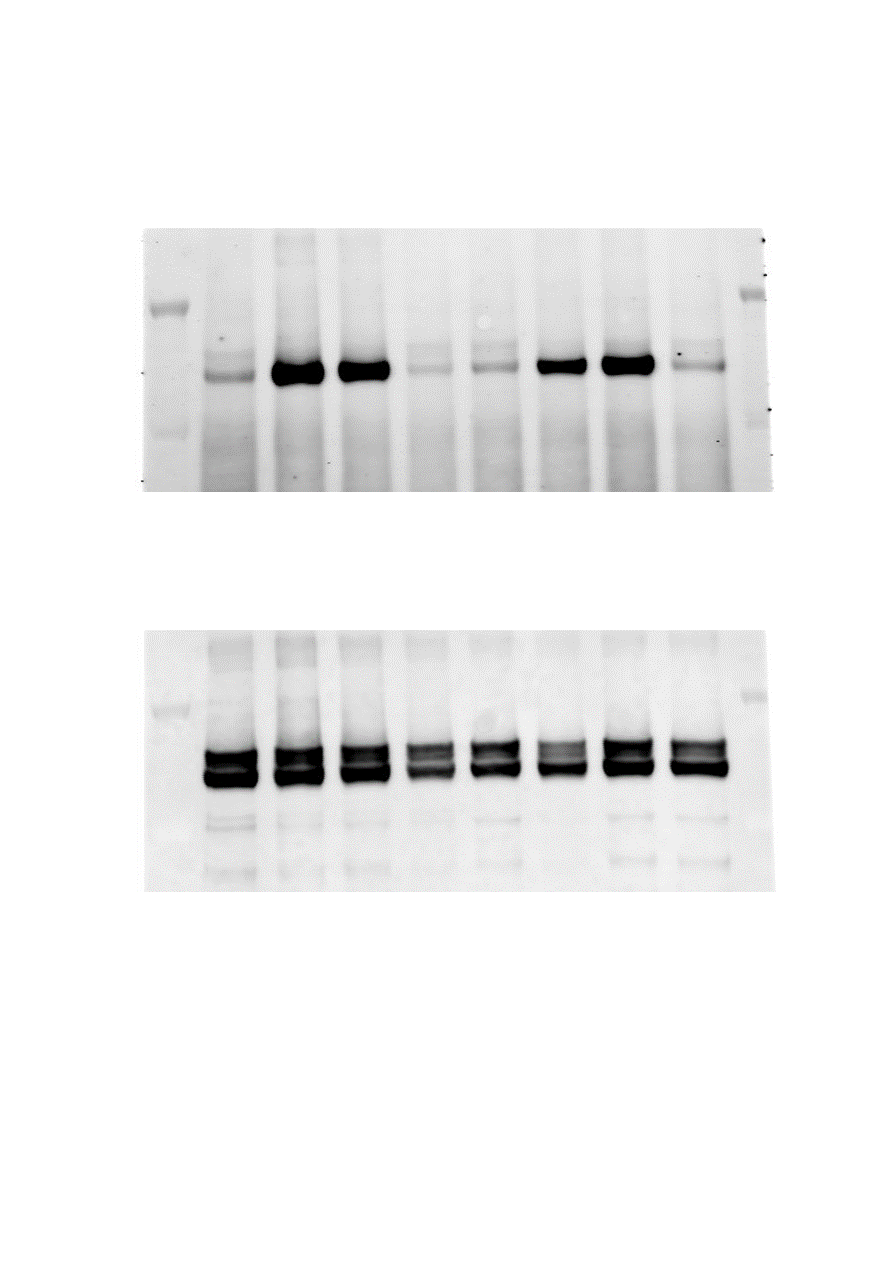

Supplement: Figure 2—source data 1. [file elife-80944-fig2-data1.zip › Resubmission Rev 2 Figure 2source data 1/Figure 2B Raw Blots.tiff]

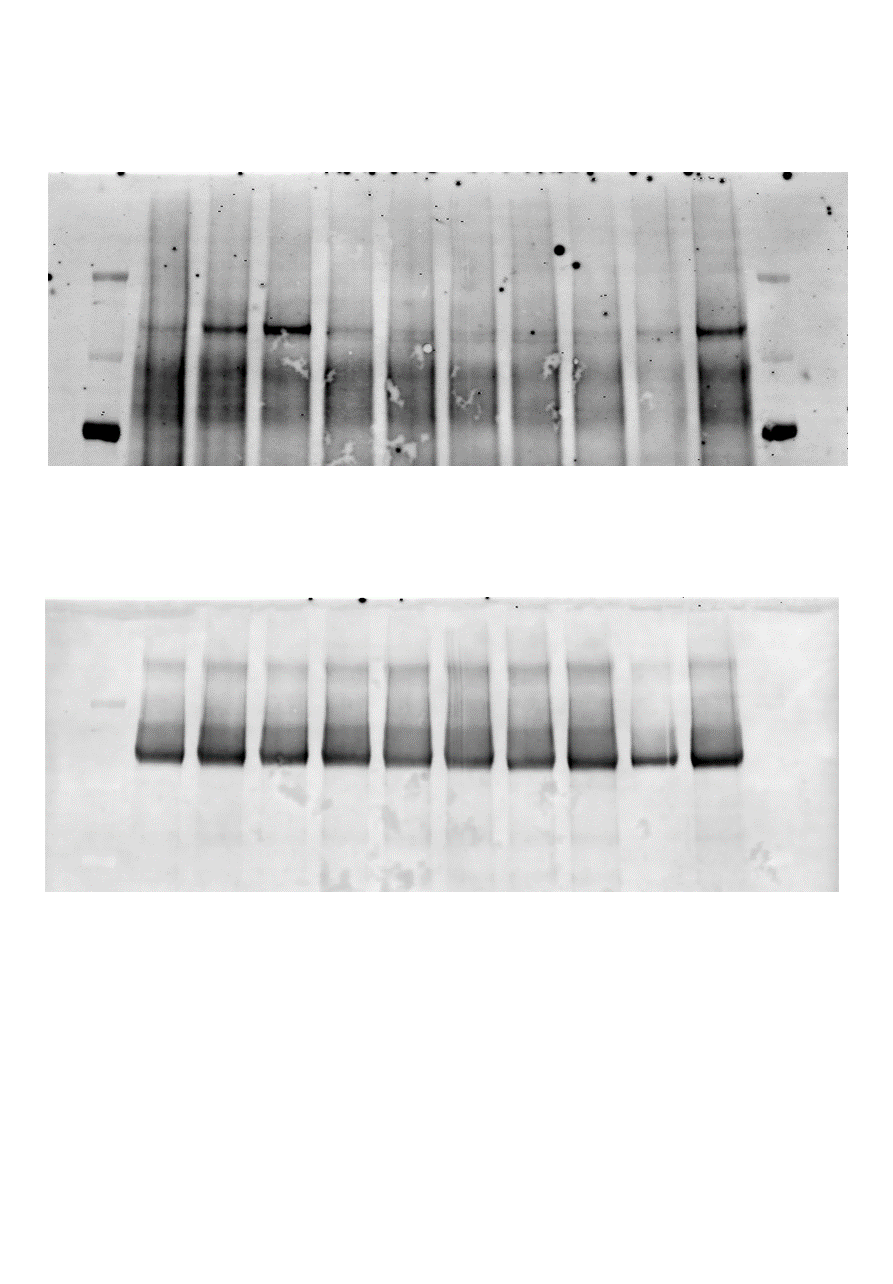

Supplement: Figure 2—source data 1. [file elife-80944-fig2-data1.zip › Resubmission Rev 2 Figure 2source data 1/Figure 2C Raw Blots.tiff]

## Slide 1
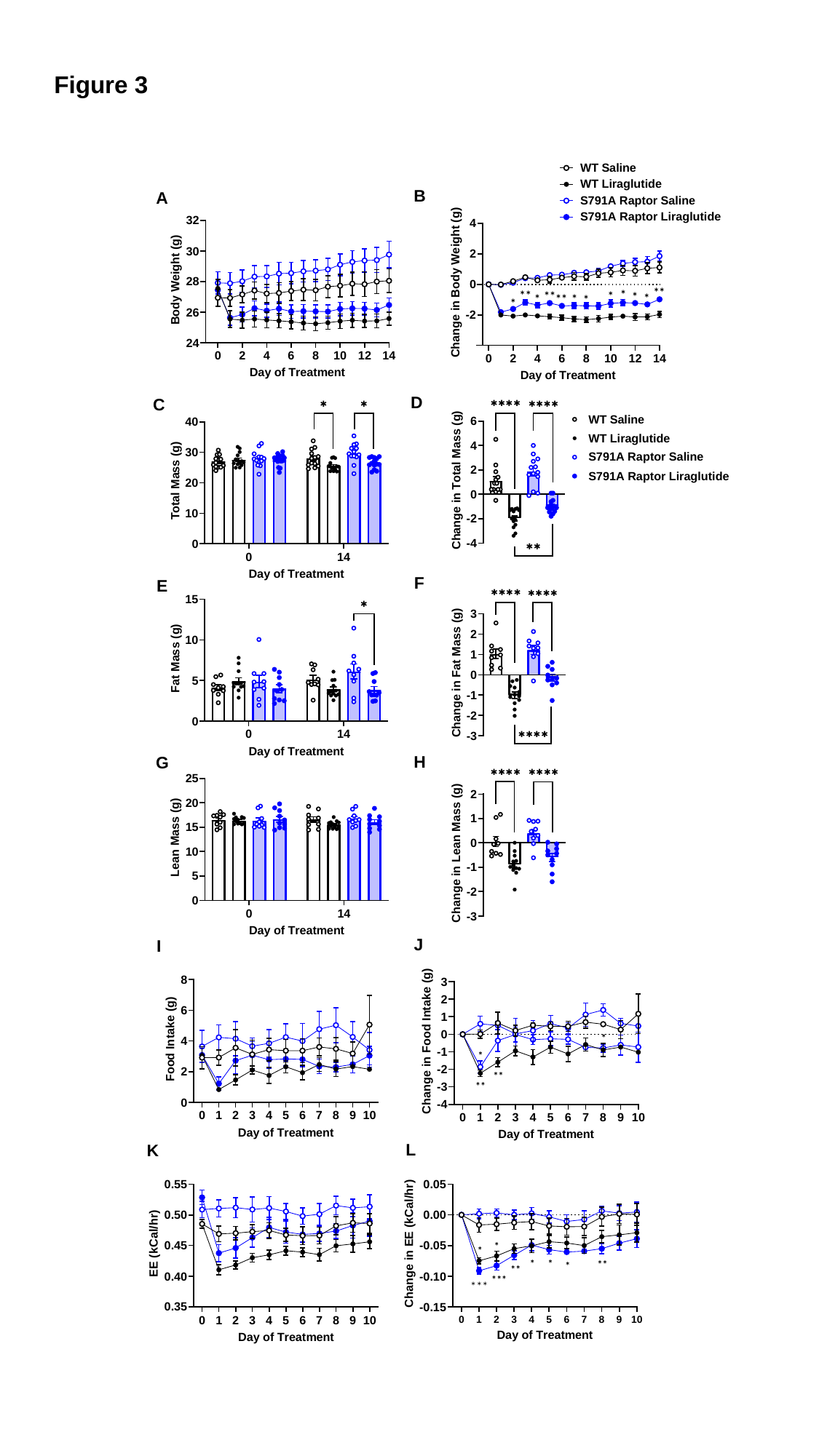

Figure 3
B
A
D
C
F
E
H
G
J
I
L
K

Supplement: Figure 3—source data 1. [file elife-80944-fig3-data1.zip › eLife PKA Manuscript Rev 2 Figure 3.pptx]

## Slide 1
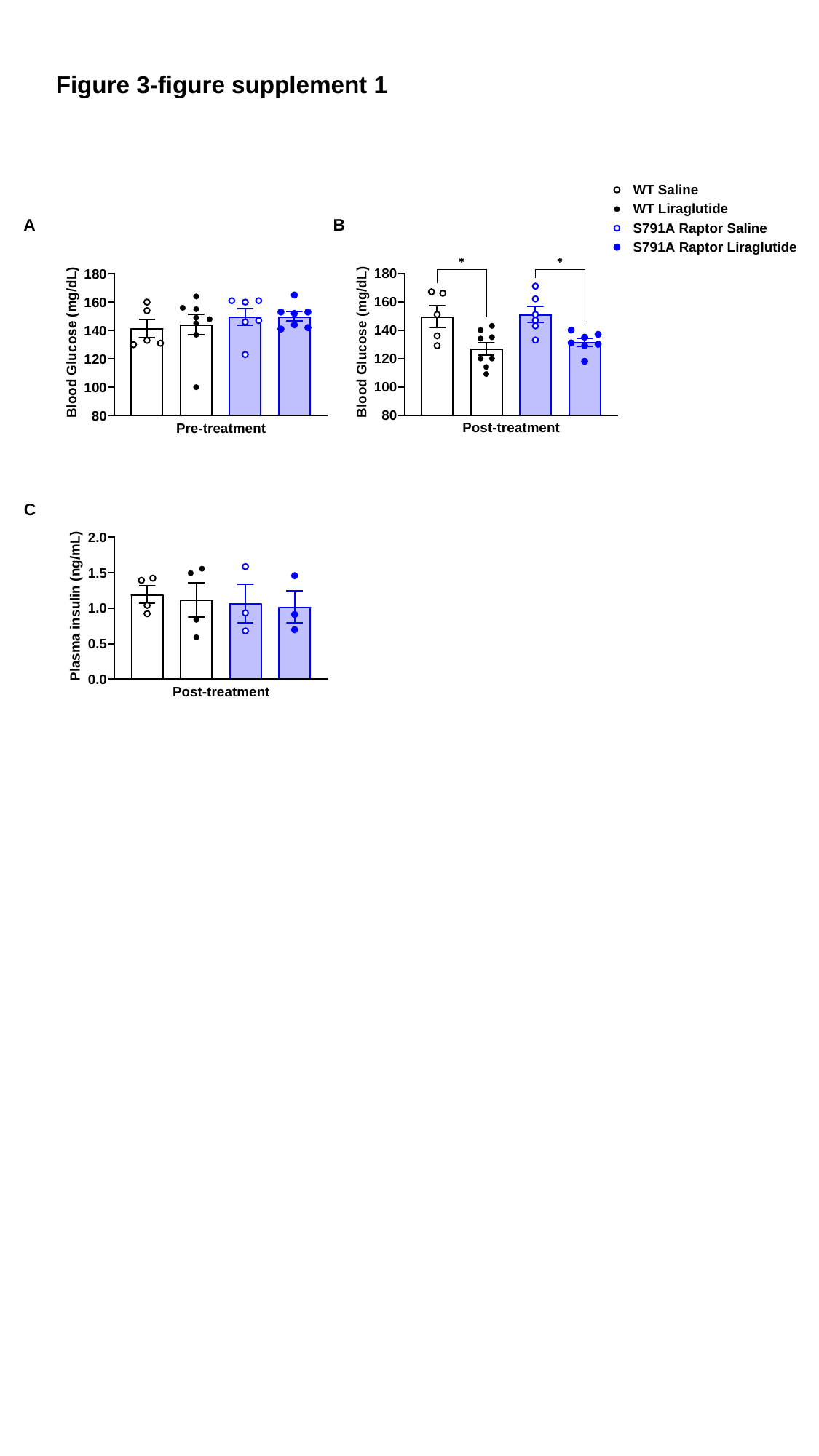

Figure 3-figure supplement 1
B
A
C

Supplement: Figure 3—figure supplement 1—source data 1. [file elife-80944-fig3-figsupp1-data1.zip › eLife PKA Manuscript Rev 2 Figure 3-figure supplement 1.pptx]

## Slide 1
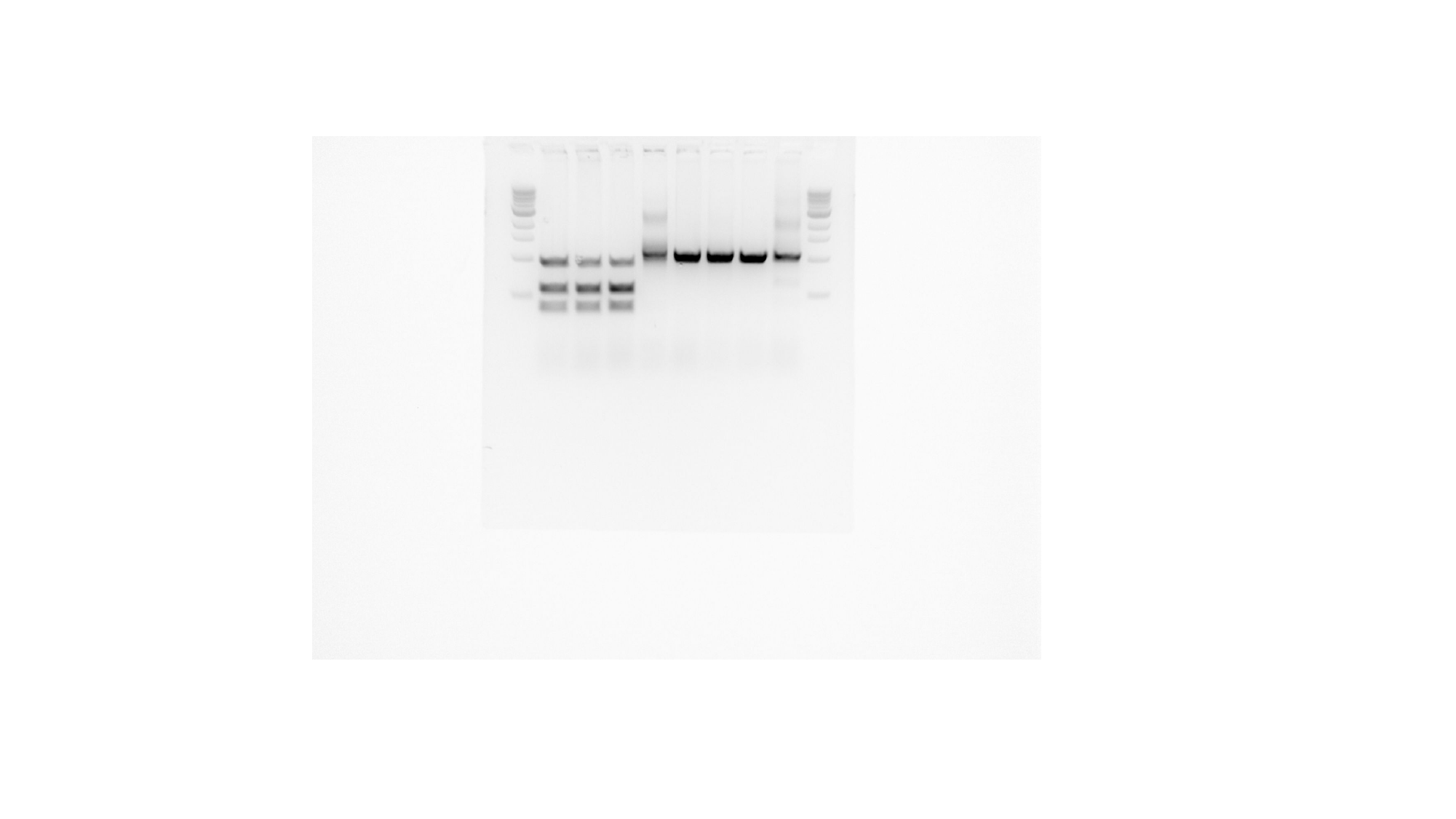

Supplement: Figure 3—figure supplement 2—source data 1. [file elife-80944-fig3-figsupp2-data1.zip › eLife PKA Manuscript Rev 2 Figure 3-figure supplement 2 Raw Blot.pptx]

## Slide 1
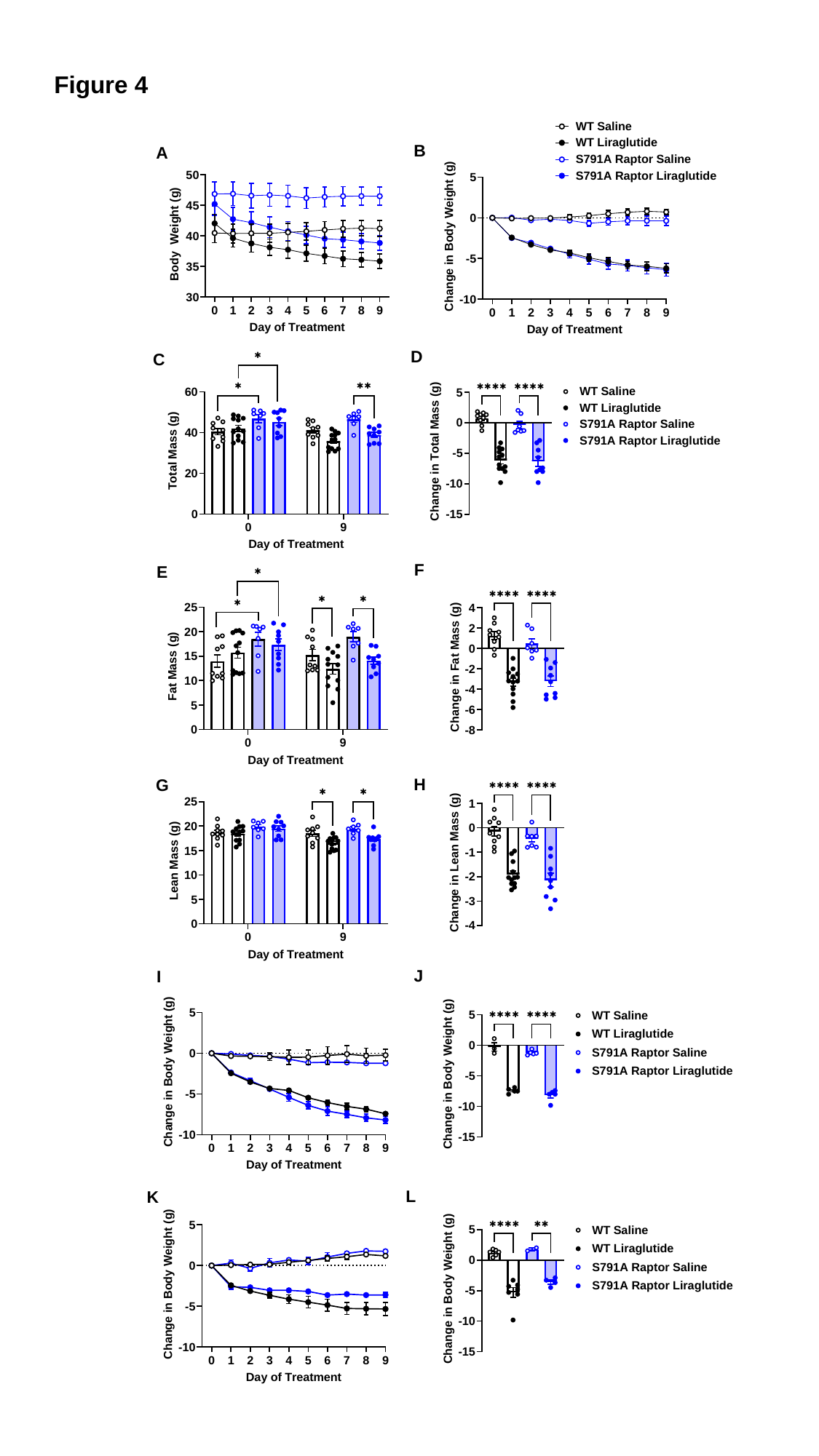

Figure 4
B
A
D
C
F
E
H
G
J
I
L
K

Supplement: Figure 4—source data 1. [file elife-80944-fig4-data1.zip › eLife PKA Manuscript Rev 2 Figure 4.pptx]

## Slide 1
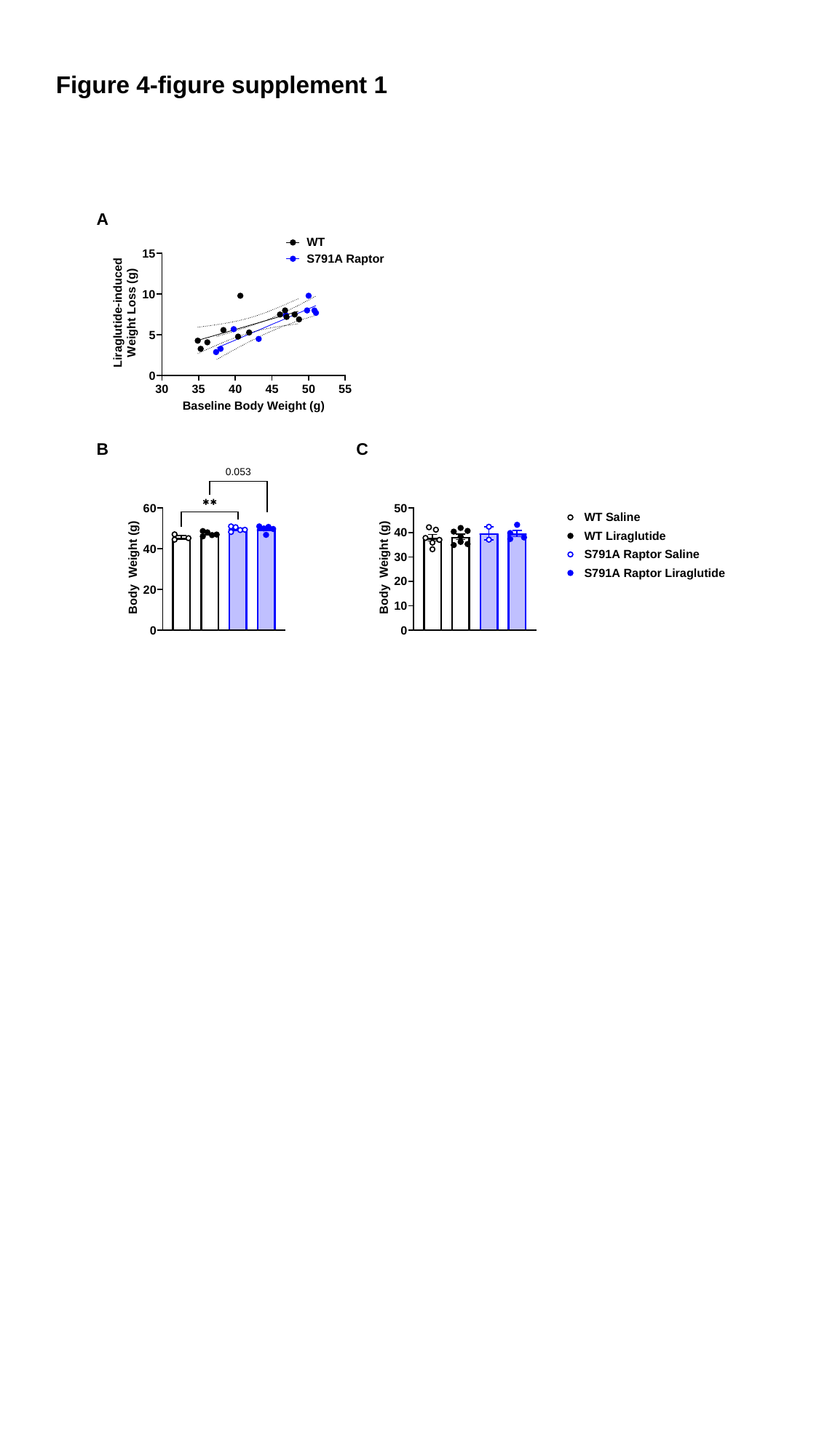

Figure 4-figure supplement 1
A
B
C

Supplement: Figure 4—figure supplement 1—source data 1. [file elife-80944-fig4-figsupp1-data1.zip › eLife PKA Manuscript Rev 2 Figure 4-figure supplement 1.pptx]

## Slide 1
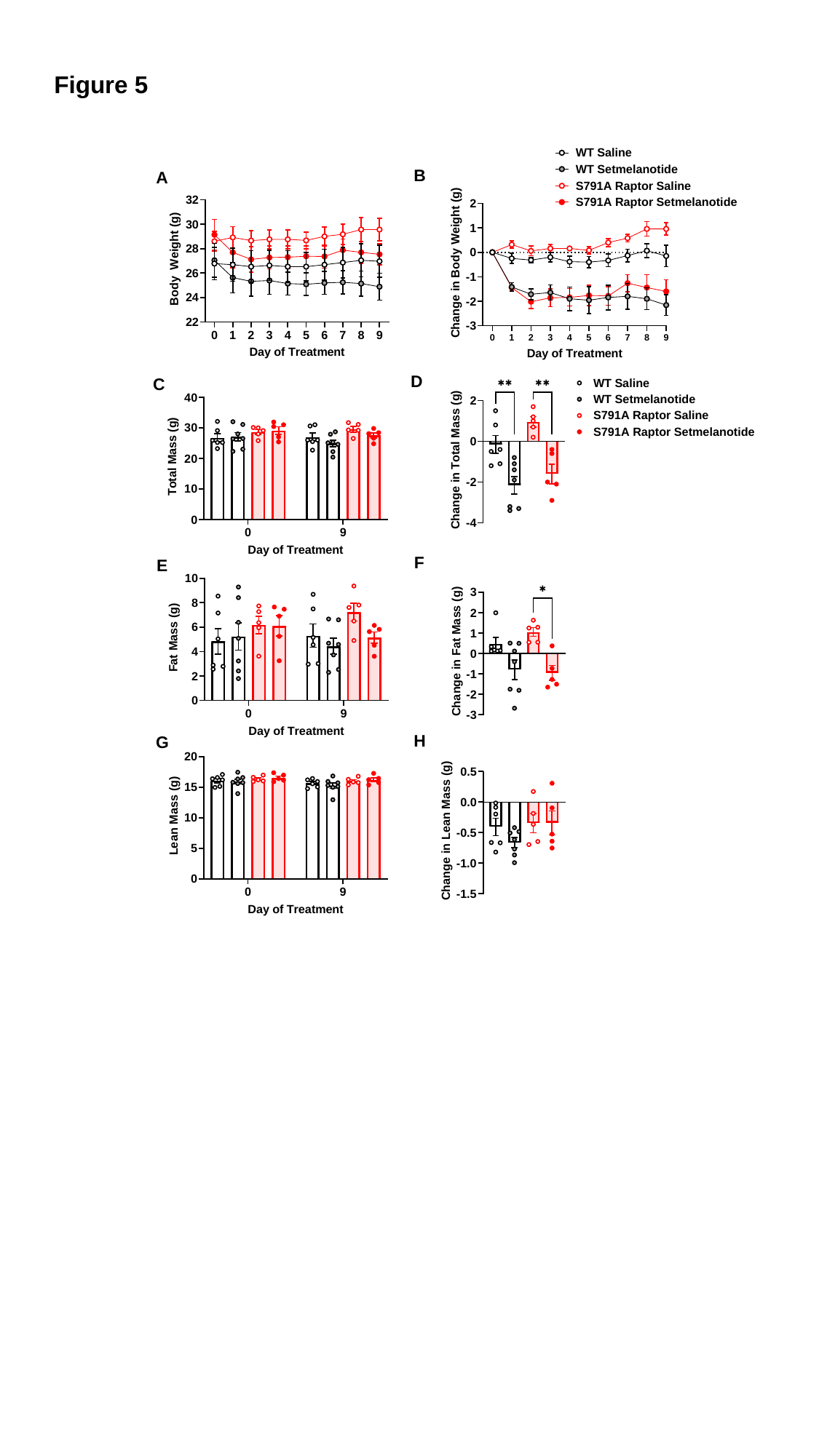

Figure 5
B
A
D
C
F
E
H
G

Supplement: Figure 5—source data 1. [file elife-80944-fig5-data1.zip › eLife PKA Manuscript Rev 2 Figure 5.pptx]
