# Supplementary material for: Glucagon-like peptide-1 receptor activation stimulates PKA-mediated phosphorylation of Raptor and this contributes to the weight loss effect of liraglutide
Source: eLife. 2023 Nov 6;12:e80944. doi: 10.7554/eLife.80944 (PMC10691799; doi:10.7554/eLife.80944)
Supplement: Figure 1—figure supplement 1—source data 1. [file elife-80944-fig1-figsupp1-data1.zip › eLife PKA Manuscript Rev 2 Figure 1-figure supplement 1.pptx]

## Slide 1
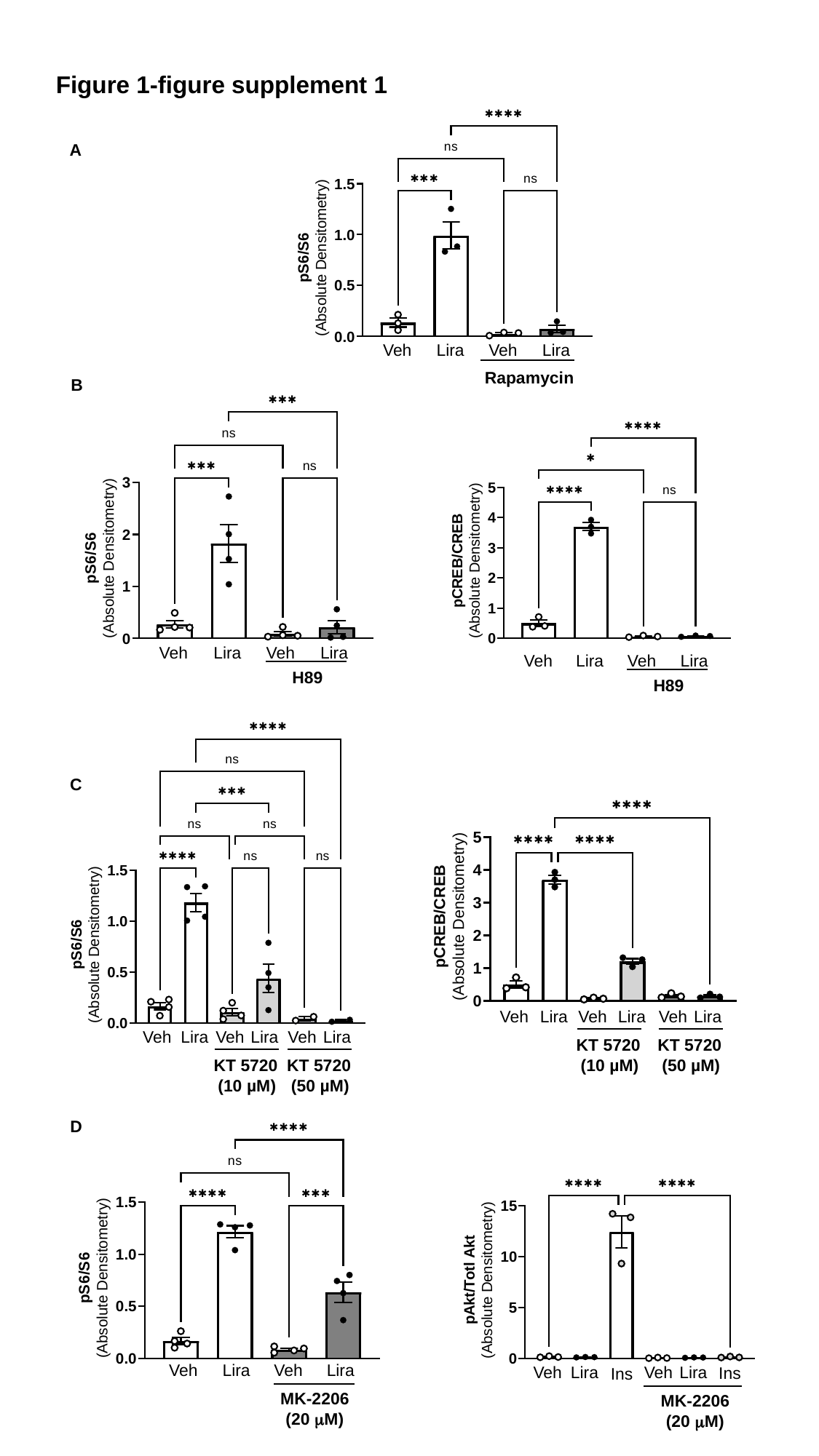

Figure 1-figure supplement 1
Veh
Lira
Veh
Lira
Rapamycin
A
B
Veh
Lira
Veh
Lira
H89
Veh
Lira
Veh
Lira
H89
Veh
Lira
Veh
Lira
Veh
Lira
KT 5720
(10 µM)
KT 5720
(50 µM)
C
Veh
Lira
Veh
Lira
Veh
Lira
KT 5720
(10 µM)
KT 5720
(50 µM)
D
Veh
Lira
Veh
Lira
Veh
Lira
Veh
Lira
Ins
Ins
MK-2206
(20 mM)
MK-2206
(20 mM)
