# Supplementary material for: Glucagon-like peptide-1 receptor activation stimulates PKA-mediated phosphorylation of Raptor and this contributes to the weight loss effect of liraglutide
Source: eLife. 2023 Nov 6;12:e80944. doi: 10.7554/eLife.80944 (PMC10691799; doi:10.7554/eLife.80944)
Supplement: Figure 1—figure supplement 2—source data 1. [file elife-80944-fig1-figsupp2-data1.zip › eLife PKA Manuscript Rev 2 Figure 1-figure supplement 2 Raw Blots.pptx]

## Slide 1
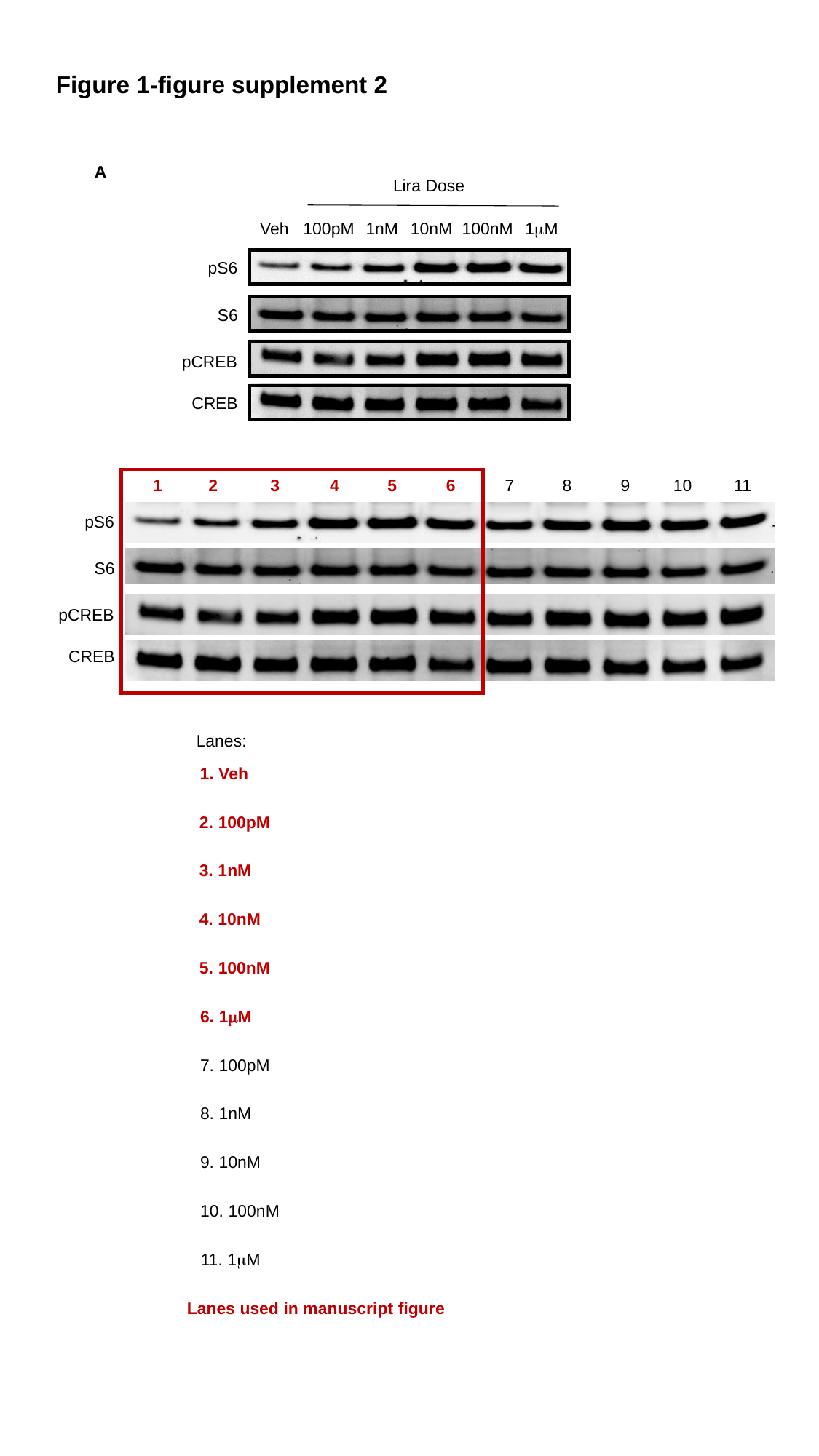

Figure 1-figure supplement 2
A
Lira Dose
Veh
100pM
1nM
10nM
100nM
1mM
pS6
S6
pCREB
CREB
1
2
3
4
5
6
7
8
9
10
11
pS6
S6
pCREB
CREB
Lanes:
1. Veh
2. 100pM
3. 1nM
4. 10nM
5. 100nM
6. 1mM
7. 100pM
8. 1nM
9. 10nM
10. 100nM
11. 1mM
Lanes used in manuscript figure

## Slide 2
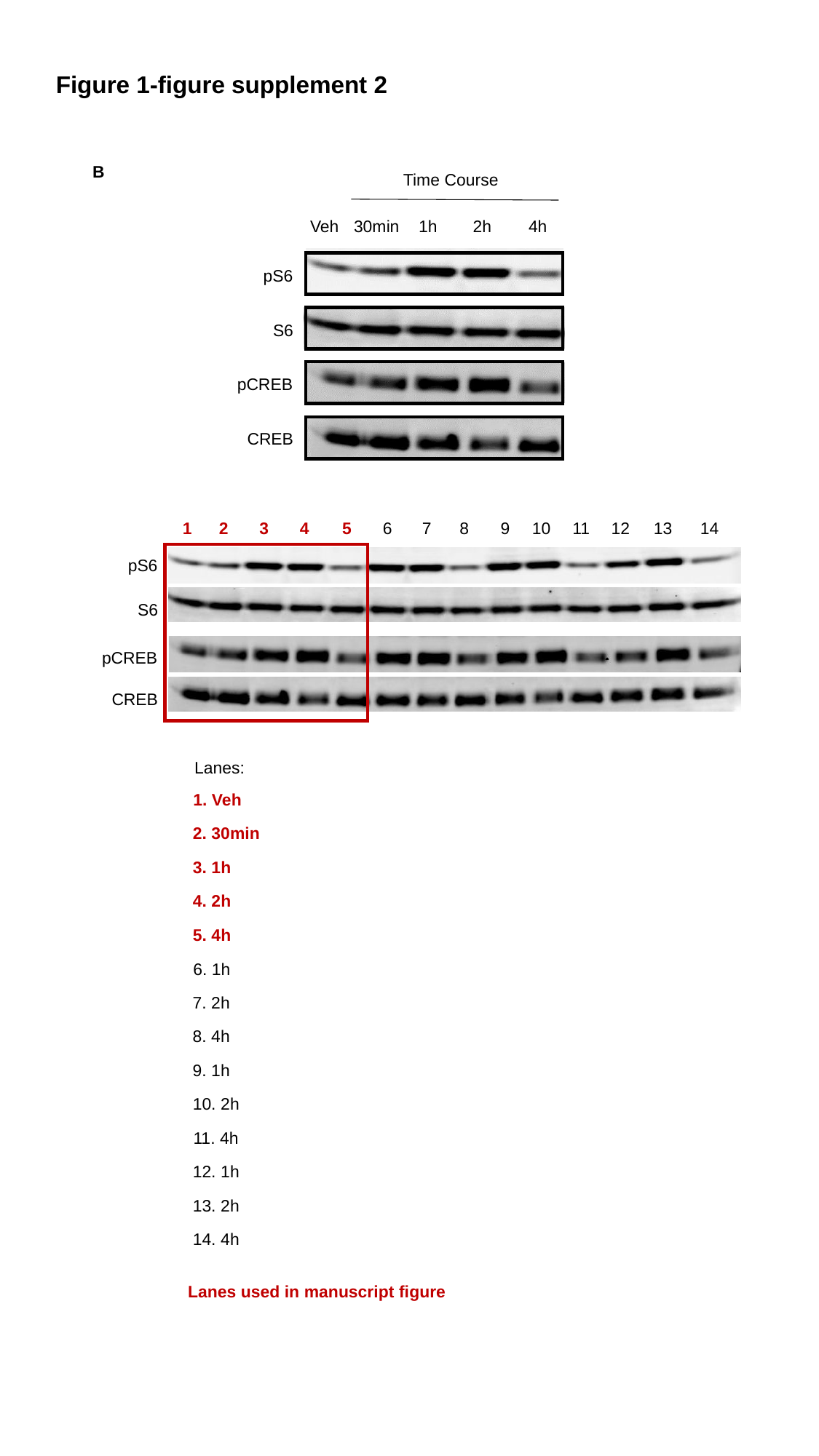

Figure 1-figure supplement 2
B
Time Course
Veh
30min
1h
2h
4h
pS6
S6
pCREB
CREB
1
2
3
4
5
6
7
8
9
10
11
12
13
14
pS6
S6
pCREB
CREB
Lanes:
1. Veh
2. 30min
3. 1h
4. 2h
5. 4h
6. 1h
7. 2h
8. 4h
9. 1h
10. 2h
11. 4h
12. 1h
13. 2h
14. 4h
Lanes used in manuscript figure
