# Supplementary material for: Glucagon-like peptide-1 receptor activation stimulates PKA-mediated phosphorylation of Raptor and this contributes to the weight loss effect of liraglutide
Source: eLife. 2023 Nov 6;12:e80944. doi: 10.7554/eLife.80944 (PMC10691799; doi:10.7554/eLife.80944)
Supplement: Figure 1—figure supplement 2—source data 1. [file elife-80944-fig1-figsupp2-data1.zip › eLife PKA Manuscript Rev 2 Figure 1-figure supplement 2.pptx]

## Slide 1
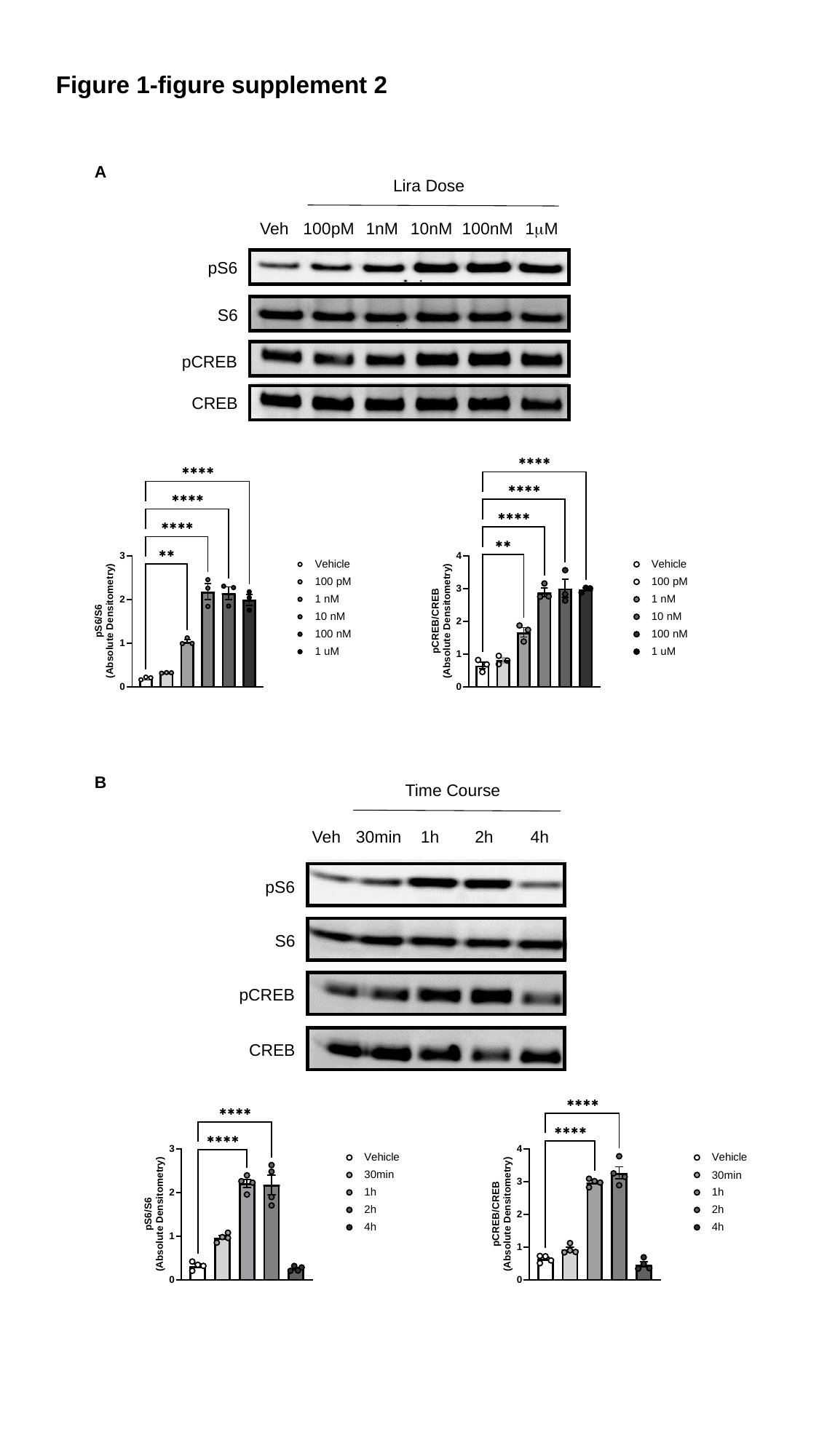

Figure 1-figure supplement 2
A
Lira Dose
Veh
100pM
1nM
10nM
100nM
1mM
pS6
S6
pCREB
CREB
B
Time Course
Veh
30min
1h
2h
4h
pS6
S6
pCREB
CREB
