# Supplementary material for: Glucagon-like peptide-1 receptor activation stimulates PKA-mediated phosphorylation of Raptor and this contributes to the weight loss effect of liraglutide
Source: eLife. 2023 Nov 6;12:e80944. doi: 10.7554/eLife.80944 (PMC10691799; doi:10.7554/eLife.80944)
Supplement: Figure 2—source data 1. [file elife-80944-fig2-data1.zip › Resubmission Rev 2 Figure 2source data 1/eLife PKA Manuscript Rev 2 Figure 2 Raw Blots.pptx]

## Slide 1
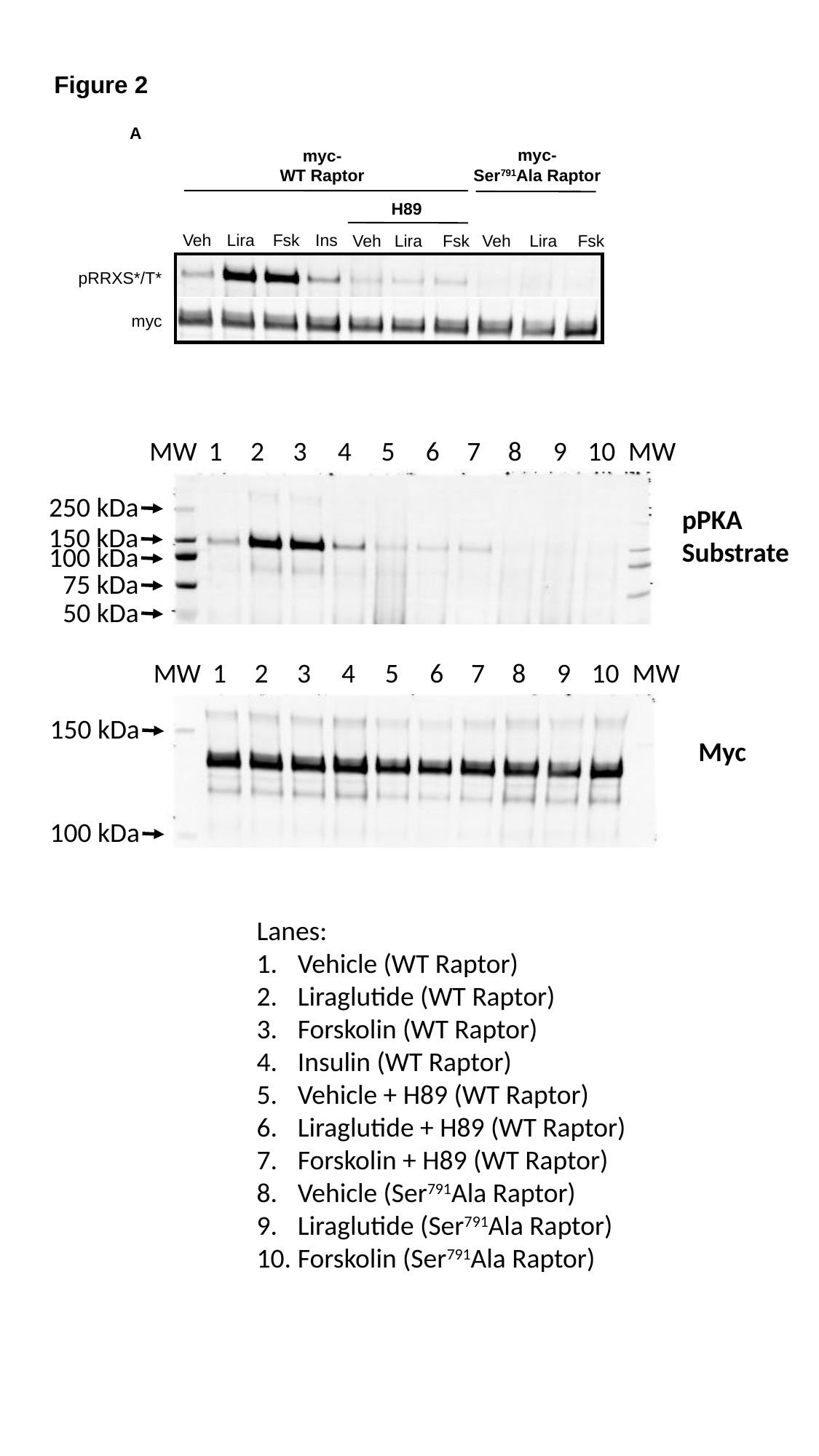

Figure 2
A
myc-
Ser791Ala Raptor
myc-
WT Raptor
H89
Veh
Lira
Fsk
Ins
Veh
Lira
Fsk
Veh
Lira
Fsk
pRRXS*/T*
myc
MW
1
2
3
4
5
6
7
8
9
10
MW
250 kDa
pPKA
Substrate
150 kDa
100 kDa
75 kDa
50 kDa
MW
1
2
3
4
5
6
7
8
9
10
MW
150 kDa
Myc
100 kDa
Lanes:
Vehicle (WT Raptor)
Liraglutide (WT Raptor)
Forskolin (WT Raptor)
Insulin (WT Raptor)
Vehicle + H89 (WT Raptor)
Liraglutide + H89 (WT Raptor)
Forskolin + H89 (WT Raptor)
Vehicle (Ser791Ala Raptor)
Liraglutide (Ser791Ala Raptor)
Forskolin (Ser791Ala Raptor)

## Slide 2
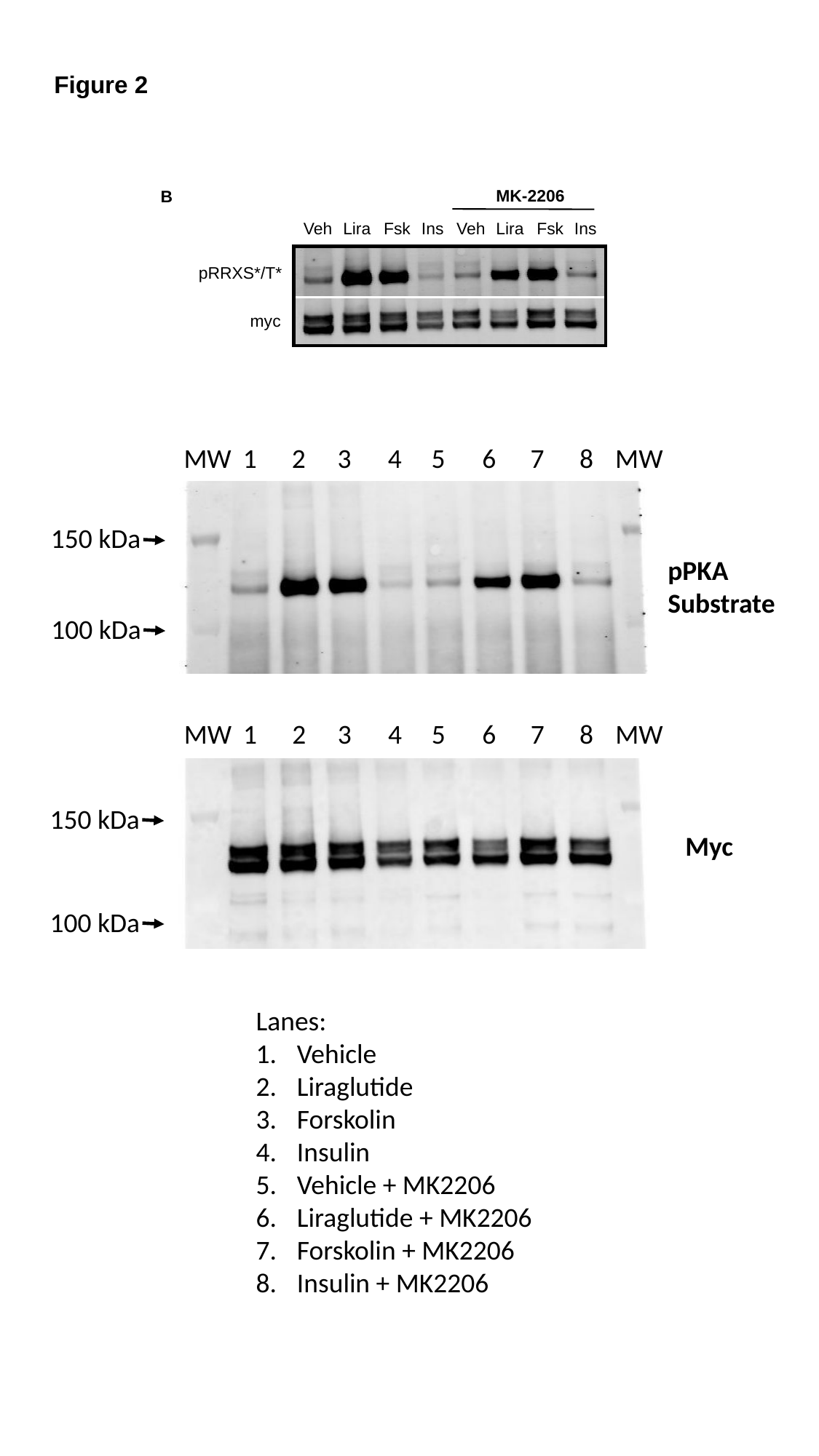

Figure 2
MK-2206
B
Veh
Lira
Fsk
Ins
Veh
Lira
Fsk
Ins
pRRXS*/T*
myc
MW
1
2
3
4
5
6
7
8
MW
150 kDa
pPKA
Substrate
100 kDa
MW
1
2
3
4
5
6
7
8
MW
150 kDa
Myc
100 kDa
Lanes:
Vehicle
Liraglutide
Forskolin
Insulin
Vehicle + MK2206
Liraglutide + MK2206
Forskolin + MK2206
Insulin + MK2206

## Slide 3
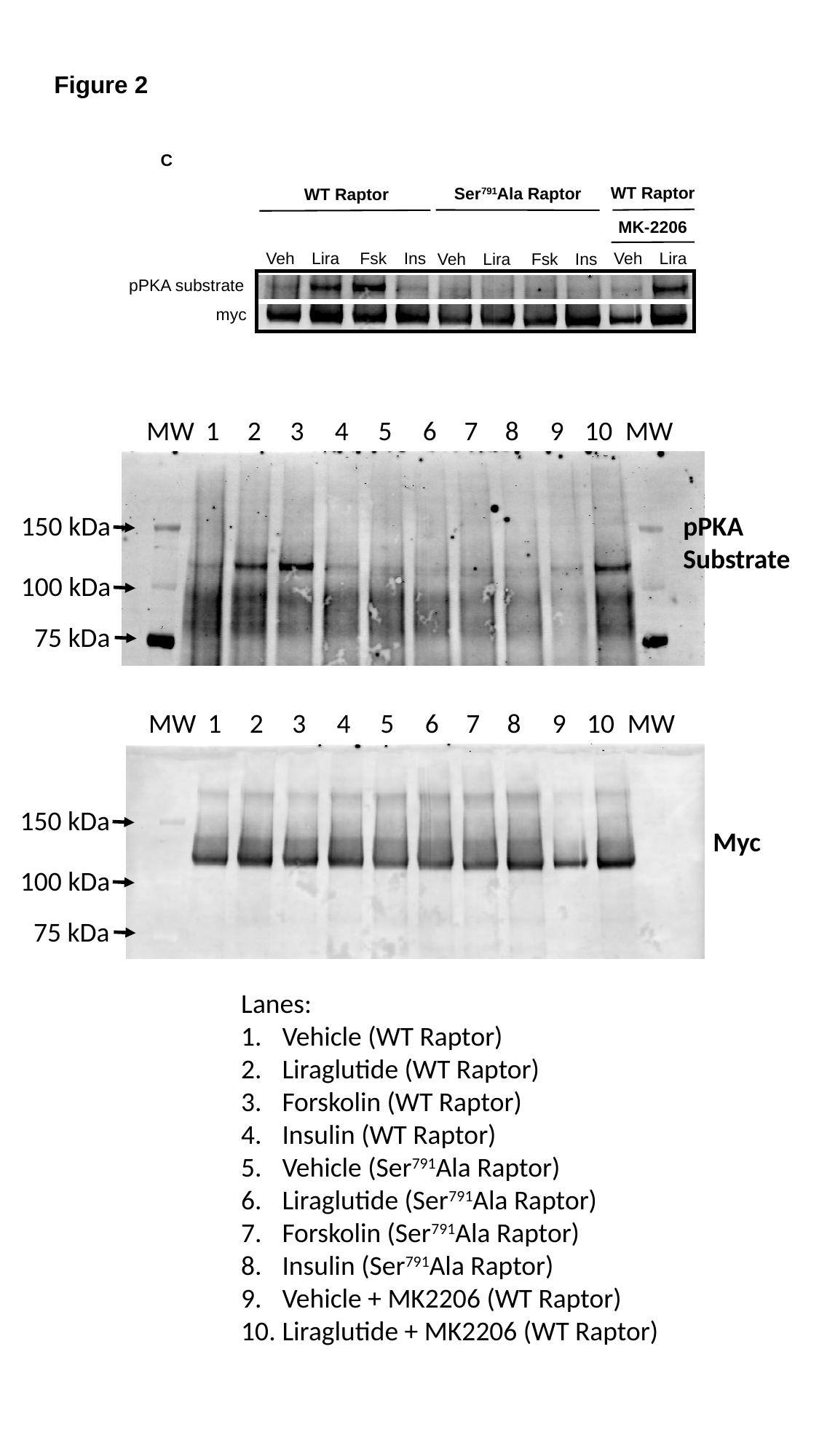

Figure 2
C
WT Raptor
Ser791Ala Raptor
WT Raptor
MK-2206
Veh
Lira
Fsk
Ins
Veh
Lira
Veh
Lira
Fsk
Ins
pPKA substrate
myc
MW
1
2
3
4
5
6
7
8
9
10
MW
pPKA
Substrate
150 kDa
100 kDa
75 kDa
MW
1
2
3
4
5
6
7
8
9
10
MW
150 kDa
Myc
100 kDa
75 kDa
Lanes:
Vehicle (WT Raptor)
Liraglutide (WT Raptor)
Forskolin (WT Raptor)
Insulin (WT Raptor)
Vehicle (Ser791Ala Raptor)
Liraglutide (Ser791Ala Raptor)
Forskolin (Ser791Ala Raptor)
Insulin (Ser791Ala Raptor)
Vehicle + MK2206 (WT Raptor)
Liraglutide + MK2206 (WT Raptor)
