# Supplementary material for: Glucagon-like peptide-1 receptor activation stimulates PKA-mediated phosphorylation of Raptor and this contributes to the weight loss effect of liraglutide
Source: eLife. 2023 Nov 6;12:e80944. doi: 10.7554/eLife.80944 (PMC10691799; doi:10.7554/eLife.80944)
Supplement: Figure 2—source data 1. [file elife-80944-fig2-data1.zip › Resubmission Rev 2 Figure 2source data 1/eLife PKA Manuscript Rev 2 Figure 2.pptx]

## Slide 1
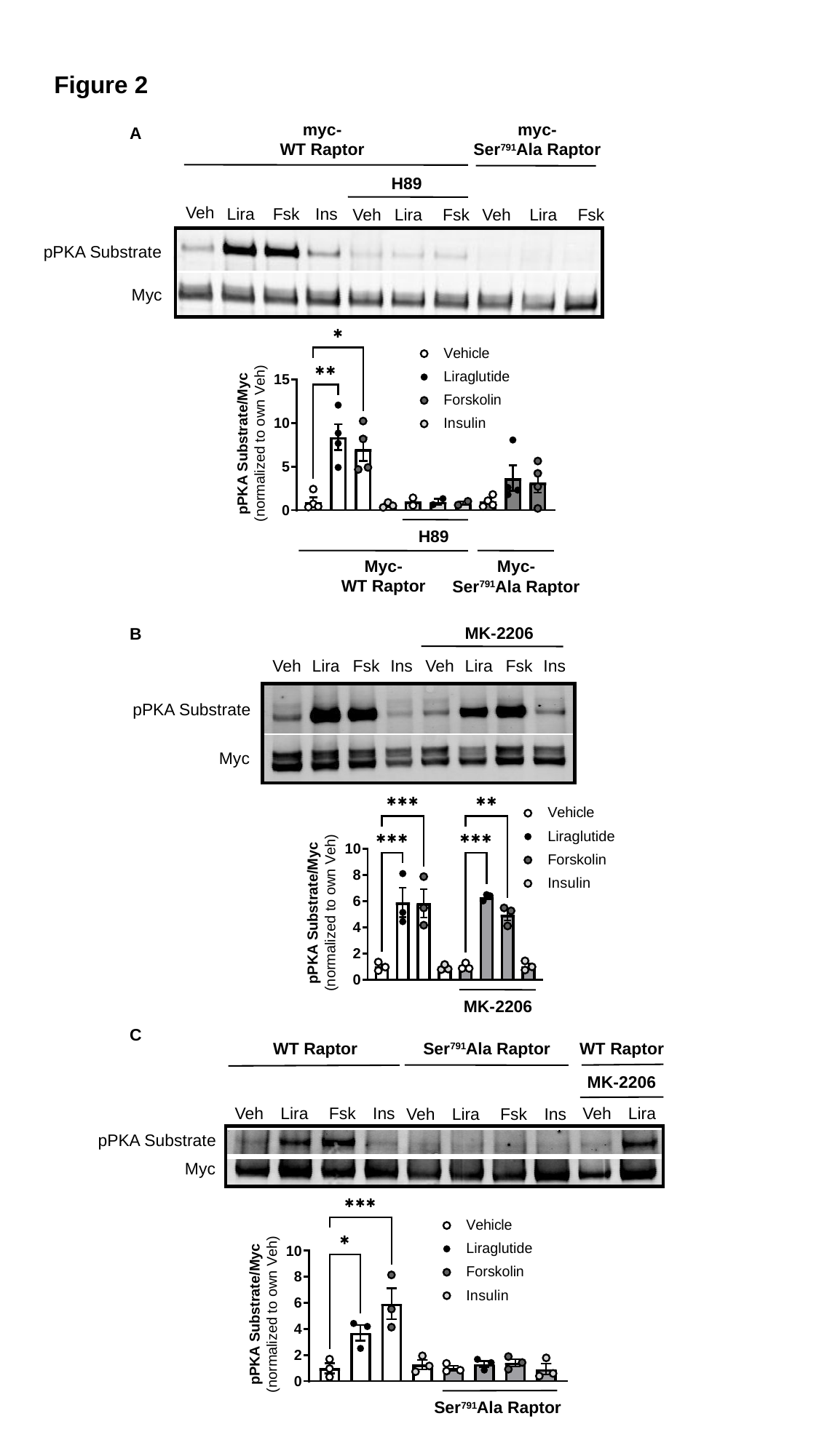

Figure 2
myc-
Ser791Ala Raptor
myc-
WT Raptor
A
H89
Veh
Lira
Fsk
Ins
Veh
Lira
Fsk
Veh
Lira
Fsk
pPKA Substrate
Myc
H89
Myc-
WT Raptor
Myc-
Ser791Ala Raptor
MK-2206
B
Veh
Lira
Fsk
Ins
Veh
Lira
Fsk
Ins
pPKA Substrate
Myc
MK-2206
C
WT Raptor
Ser791Ala Raptor
WT Raptor
MK-2206
Veh
Lira
Fsk
Ins
Veh
Lira
Veh
Lira
Fsk
Ins
pPKA Substrate
Myc
Ser791Ala Raptor
