# Supplementary material for: Glucagon-like peptide-1 receptor activation stimulates PKA-mediated phosphorylation of Raptor and this contributes to the weight loss effect of liraglutide
Source: eLife. 2023 Nov 6;12:e80944. doi: 10.7554/eLife.80944 (PMC10691799; doi:10.7554/eLife.80944)
Supplement: Figure 3—figure supplement 2—source data 1. [file elife-80944-fig3-figsupp2-data1.zip › eLife PKA Manuscript Rev 2 Figure 3-figure supplement 2.pptx]

## Slide 1
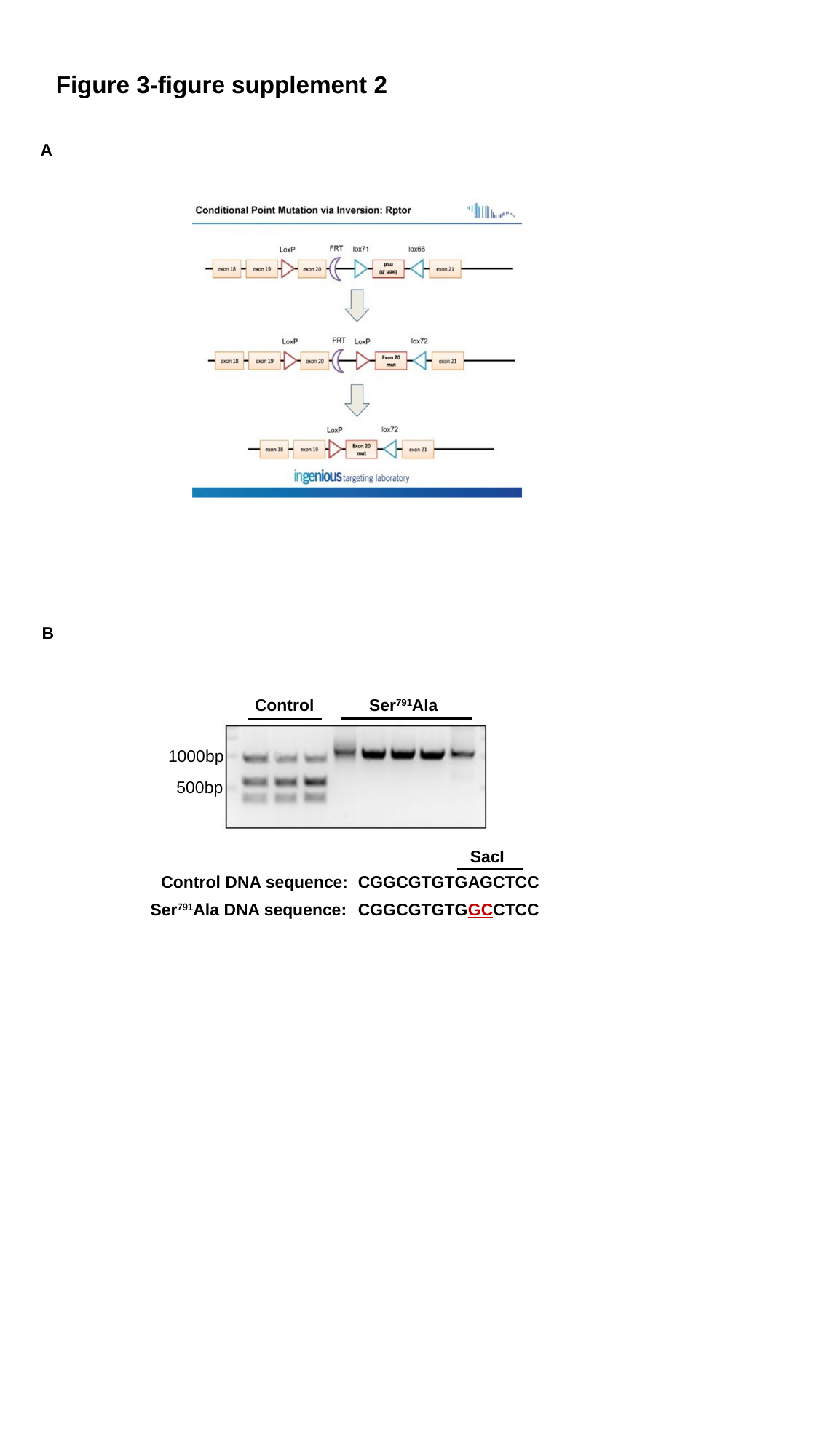

Figure 3-figure supplement 2
A
B
Control
Ser791Ala
1000bp
500bp
SacI
Control DNA sequence:
CGGCGTGTGAGCTCC
Ser791Ala DNA sequence:
CGGCGTGTGGCCTCC
